# Brain dynamics of crosslinguistic interference resolution in Spanish–English bilinguals with and without aphasia

**DOI:** 10.1017/S1366728925100461

**Published:** 2026-08

**Authors:** Katherine Diane Andrade, Henrike K. Blumenfeld, Stéphanie Kathleen Riès

**Affiliations:** 1 Joint Doctoral Program in Language and Communicative Disorders, San Diego State University/University of California San Diego, La Jolla, CA, USA; 2 School of Speech, Language and Hearing Sciences, San Diego State University, San Diego, CA, USA

**Keywords:** bilingualism, crosslinguistic interference, language production, stroke-induced aphasia, EEG

## Abstract

Bilinguals simultaneously activate both languages during word retrieval. False cognates, words overlapping in form but not meaning across languages, typically trigger crosslinguistic interference relative to non-cognates. Crosslinguistic interference resolution can be impaired in bilinguals with stroke-induced aphasia, yet little is known about the neural dynamics supporting these interference resolution processes. We recorded scalp electroencephalography in 21 age-matched controls and five bilinguals with aphasia participating in a picture-word interference paradigm eliciting crosslinguistic interference and a nonlinguistic spatial Stroop task. Bilinguals with aphasia showed lower performance than age-matched controls and crosslinguistic interference was present across both groups. A medial frontal component peaking around 400 ms post stimulus presentation was present in controls across tasks but was absent in the linguistic task in bilinguals with aphasia. This suggests that while bilinguals typically engage the medial frontal cortex to resolve crosslinguistic interference, this mechanism is disrupted in bilinguals with aphasia.

## Highlights

- We investigated how bilinguals resolve crosslinguistic interference.
- Spanish–English bilinguals with and without aphasia named pictures.
- Performance is lower if false cognates versus unrelated distractors have to be ignored.
- Controls showed a medial-frontal ERP sensitive to crosslinguistic interference.
- The underlying control mechanism appears disrupted in bilinguals with aphasia.

## 1. Introduction

The growing prevalence of bilingual speakers in the United States and worldwide has generated great interest in the linguistic and cognitive abilities associated with bilingualism. Bilinguals routinely activate both languages in parallel while listening and preparing to speak (e.g., Colomé, 2001; Dijkstra, 2005; Kroll et al., 2006, Kroll et al., 2008; Marian & Spivey, 2003; Abutalebi & Green, 2007; Dijkstra & Heuven, 1998). Current models of bilingual processing generally agree that lexical representations from both languages are active; however, some models suggest that crosslinguistically activated words compete for selection (e.g., Costa, 2005; Costa et al., 1999; Roelofs et al., 2013) while others have proposed non-competitive accounts during lexical selection (e.g., Blanco-Elorrieta & Caramazza, 2021; Declerck & Philipp, 2015; La Heij, 2005; Poulisse & Bongaerts, 1994). Further, some researchers have proposed that an inhibitory process is needed to modulate the activation of the nontarget language in order to resolve conflict between languages (e.g., Abutalebi & Green, 2016; Green & Abutalebi, 2013; but see Blanco-Elorrieta & Caramazza, 2021). Crosslinguistic interaction refers to the influence that knowledge of one language has on an individual’s use of another language and the underlying processing mechanisms. Parallel activation at any level of language production (i.e., semantic, lexical, phonological) can result in both facilitation (with cognates, e.g., the knowledge of English *lamp* may boost performance on Spanish *lámpara*, Goral et al., 2006; Roberts & Deslauriers, 1999) or interference (with false cognates, e.g., the knowledge of English *blue* may slow performance on Dutch *blut*, meaning *broke* during word reading in Dutch, van Heuven et al., 2008). Specifically, false cognates activate overlapping phonetic and orthographic representations but separate semantic representations across languages, which can cause interference during the lexical retrieval process (e.g., Dijkstra & Heuven, 1998; Shook & Marian, 2013; Blanco-Elorrieta & Caramazza, 2021). Previous studies have reported lower accuracy rates and longer reaction times for false cognate processing compared to non-cognate words in bilinguals (e.g., van Heuven et al., 2008; Vanlangendonck et al., 2020; Von Studnitz & Green, 2002). Yet little is known about the neural dynamics supporting such crosslinguistic interference resolution. In addition to examining such neural processes in unimpaired bilinguals, the inclusion of individuals with stroke-induced aphasia is valuable to understand the neural mechanisms of crosslinguistic bilingual processing that may be impacted in bilinguals with aphasia.

Aphasia is an acquired language impairment that predominantly occurs following left hemisphere stroke-induced brain damage. More than two million people in the United States suffer from post-stroke aphasia (American Speech-Language-Hearing Association, 2024). In bilingual persons with stroke-induced aphasia, cognitive and linguistic deficits can impact each language, but also how the two languages interact with each other. The interaction between L1 and L2 processing in bilinguals with aphasia has been studied using behavioral, computational and electrophysiological methods (Khachatryan et al., 2018; Kiran et al., 2014; Kiran & Iakupova, 2011; Siyambalapitiya et al., 2013). These studies showed that participant characteristics including lesion location and proficiency levels influence the degree of language impairment in bilinguals with aphasia.

Bilinguals have been argued to rely on cognitive control processes to select and produce the correct word from their lexicon in the context-appropriate language (e.g., Green, 1998; Green & Abutalebi, 2013). Cognitive control refers to the mechanisms that allow us to inhibit, monitor and control automatic behavioral responses to support adaptive goal-directed behavior (see, e.g., Miller & Cohen, 2001; Duncan, 2010; Mackie et al., 2013). In bilingualism, cognitive control may operate in the form of an external mechanism that helps boost the activation of representations in the target language to select the correct lexical representation for the given linguistic environment (Blanco-Elorrieta & Caramazza, 2021), similar to the booster mechanism proposed by Oppenheim et al. (2010) in within-language contexts. The impact of bilingual proficiency on performance during both linguistic and nonlinguistic control tasks has led researchers to propose a mechanism for control that develops as skills in L2 are acquired (e.g., Green & Abutalebi, 2013) and can transfer to more general executive control processes (e.g., Bialystok et al., 2004). In both monolinguals and bilinguals with aphasia, the rate of spreading activation, the maintaining of the activation of a node and the selection threshold during the process of word retrieval have been proposed to be impaired (Silkes & Anjum, 2021; Green & Abutalebi, 2013; Nozari & Dell, 2013). Aphasia affects the ability to decode or encode linguistic information across multiple modalities including production, comprehension, reading and writing (Darley, 1982), language-supportive cognitive processes (e.g., Martin et al., 2008; Martin & Reilly, 2012; Murray, 2012), but especially word retrieval mechanisms (Goodglass & Kaplan, 1983). Despite the increasing prevalence of bilingualism and the critical role cognitive control mechanisms are thought to play in word retrieval, how these processes may be impacted in bilinguals with aphasia has been scarcely studied (Faroqi-Shah et al., 2018; Khachatryan et al., 2018; Nair et al., 2021; Van der Linden et al., 2018).

Compared to unimpaired bilinguals, bilinguals with aphasia show a reduction in performance in tasks that elicit conflict compared to tasks where no conflict is evoked (Dash & Kar, 2014; Gray & Kiran, 2015; Green, 2011; Green et al., 2010; Mooijman et al., 2022). For example, during the arrow version of the Eriksen flanker task (Eriksen & Eriksen, 1974; Stoffels & van der Molen, 1988), when the target arrow’s direction is incongruent to that of the surrounding arrows, individuals with aphasia will respond more slowly compared to congruent stimuli (Green et al., 2010). These cognitive resources that bilinguals may leverage for lexical selection in the presence of crosslinguistic competition are thought to be at least in part domain-general (Dash & Kar, 2014). Neuroimaging studies have demonstrated the role of the anterior cingulate cortex (ACC) and pre-supplementary motor area (pre-SMA) in the detection and management of conflict between languages (Abutalebi et al., 2007; Abutalebi & Green, 2008; Abutalebi & Green, 2016; Hernandez et al., 2000; van Heuven et al., 2008) and nonlinguistic switching tasks (Garbin et al., 2010). Although neuroimaging measures are well-positioned to elucidate whether a shared neural network exists between linguistic and cognitive control skills in bilinguals, additional methodologies are required to understand when functional overlap across linguistic and nonlinguistic processes may occur (Coderre et al., 2015; Garbin et al., 2010; van Heuven & Dijkstra, 2010).

The time course of lexical access and selection in speech production and nonlinguistic control have been studied using behavioral and electrophysiological tasks across a variety of paradigms including language switching (Jackson et al., 2001), go no-go (Rodriguez-Fornells et al., 2006) and negative priming paradigms (Dash & Kar, 2020). A recent study (Mendoza et al., 2021) implemented a picture-word matching and the arrow version of the Eriksen flanker task (Eriksen & Eriksen, 1974; Stoffels & van der Molen, 1988) using Laplacian-transformed ERPs to investigate crosslinguistic interference resolution mechanisms in college-age Spanish–English bilinguals. EEG results revealed a medial frontal component peaking prior to electromyographic (EMG) onset leading to a motor response in both the picture-word matching and the flanker task. Importantly, this medial frontal component had previously been associated with response selection outside of language as it is absent when no choice between responses needs to be made (Vidal et al., 2011). In addition, a left prefrontal potential was observed peaking at around 200 ms prior to EMG onset in the linguistic task but was absent in the nonlinguistic task. These results suggested a partial overlap between cognitive control processes across domains. While these results are informative, the picture-matching paradigm required participants to respond by pressing buttons as opposed to overtly articulating their response. The processes involved may therefore be different than those involved when speaking. Finally, the study was focused on young control participants and did not investigate how the processes of interest may be impacted by age or by stroke-induced aphasia.

### 1.1. Current study

The purpose of the current study is to investigate the neurobiological bases and nature of crosslinguistic interference resolution in Spanish–English bilingual speakers with and without aphasia caused by left hemisphere stroke-induced brain lesions. We recorded scalp EEG in Spanish–English adult control participants and Spanish–English bilingual persons with aphasia across two tasks: an expressive picture-word interference (PWI) task (e.g., Hoshino & Thierry, 2011) and a nonlinguistic Stroop task (Blumenfeld & Marian, 2011, 2014; Hilchey & Klein, 2011; Liu et al., 2004). In the PWI task, participants named a picture while ignoring a concurrently presented superimposed word. The PWI paradigm included incongruent trials using false cognates, congruent identity trials where picture and words matched and a neutral control condition with unrelated picture and word combinations. In order to examine the potential overlap between brain dynamics of linguistic and nonlinguistic cognitive control in bilinguals, participants also completed the arrow version of the Stroop task to reduce the linguistic component as much as possible in order to examine cognitive control processes as engaged outside of language (Bialystok, 2006; Blumenfeld & Marian, 2011, 2013; Capizzi et al., 2017).

The purpose of the study was twofold. We tested whether (1) a similar medial frontal cognitive control mechanism is used to resolve conflict in the face of linguistic and nonlinguistic interference; and (2) whether this medial-frontal control mechanism engaged in crosslinguistic interference resolution is impaired in bilinguals with left-hemisphere stroke-induced aphasia. First, we hypothesized that the presence of false cognate distractor words would elicit the expected crosslinguistic interference in the form of slower reaction times and lower accuracy rates compared to unrelated distractor words. If the control mechanism is impaired in bilinguals with aphasia, then this effect should be larger compared to the control participants. In addition, we hypothesize that the medial frontal component will be larger in amplitude in the incongruent as compared to the congruent conditions in the linguistic and nonlinguistic tasks in unimpaired adult controls (as seen in Mendoza et al., 2021). If the control mechanism is impaired in bilinguals with aphasia, we predict that this medial frontal component will be absent or smaller in bilingual individuals with aphasia.

## 2. Method

### 2.1. Participants

Participants were recruited from San Diego County’s community. Our participants were 11 right-handed Spanish–English bilingual persons with aphasia (*mean age* = 56.7 years; *range* = 38–78; *SD* = 12, 6 males) and 26 Spanish–English bilingual adult control participants who matched the age range and bilingual profiles of the participants with aphasia (*mean age* = 51.7 years; *range* = 32–71; *SD* = 9.5, 7 males). Eligibility criteria required participants to be 30–80 years old, right-handed, have normal or corrected-to-normal vision and have no history of learning disabilities, substance abuse or psychiatric or neurological conditions (other than stroke-induced aphasia).

Participation in this study depended on proficiency in only the targeted languages (Spanish–English), excluding individuals who reported expressive or receptive proficiency for either language of less than four across the 11-point proficiency scales of the *Language Experience and Proficiency Questionnaire* (LEAP-Q, Marian et al., 2007), or who reported multilingual skills. Furthermore, all bilingual persons with aphasia reported having had a left hemisphere stroke more than six months prior to testing and had no severe expressive impairment or apraxia of speech that would limit participation in the current language production study. Six control participants and six bilingual persons with aphasia were excluded from the analyses due to the presence of one or more ineligibility criteria that was revealed after recruitment, attrition across participation sessions or technical issues, which led to a total sample size of 21 control participants (*mean age =* 52.2 years*, SD* = 9.7) and 5 bilingual persons with aphasia (*mean age* = 57.6 years, *SD* = 15.7). Age-matched controls and bilingual persons with aphasia did not differ in age range, *t*(5) = −.73, *p* = .499. See Table 1 for these participants’ demographic information, brain lesion location, language experience and proficiency profiles.

**Table 1.** Participant demographics Table 1. Long description.

	Controls (n = 21)	Bilingual persons with aphasia (n = 5)				
	Mean (SD; range)	PT1	PT4	PT5	PT7	PT9
Age	52.2 (9.7; 32–71)	38	77	47	66	59
Gender	16F; 5M	F	M	F	F	F
Years of education	16 (4.9;4–29.8)	13	18	16.5	14	14
Age of acquisition – **Spanish**	1.0 (2; 0–7)	2	0	1	5	0
Age of acquisition – **English**	11.5 (8.8; 0–34)	11	7	5	0	16
*Current* language dominance composite score (negative values = English dominance; positive values = Spanish dominance)	−.065 (.32; −.5 to .6)	.35	−.60	−.07	−.36	−.20
*Current* *Spanish* self-rated proficiency	8.7 (1.5; 5.7–10.0)	6.7	2.7	4.7	6.3	8
*Pre-stroke Spanish* self-rated proficiency	NA	DNT	8.7	10	8	9
*Current English* self-rated proficiency	8.2 (1.7; 4.0–10.0)	3.7	6.7	4.0	9	8
*Pre-stroke English* self-rated proficiency	NA	DNT	10	10	10	9
*Current Spanish* Exposure (proportion of the time)	.41 (.25; .1–.9)	.7	.1	.4	.05	.31
Current *English* Exposure (proportion of the time)	.59 (.25; .1–.9)	.3	.9	.6	.95	.7
Handedness	Right	Right	Right	Right	Right	Right
Time since stroke (mo.)	NA	-	16	24	55	64
Aphasia severity	NA	Mild mixed expressive and receptive	Mild mixed expressive and receptive	Mild mixed expressive and receptive	Moderate mixed expressive and receptive	Mild mixed expressive and receptive
Cognition severity	NA	Moderate cognitive impairment	Moderate cognitive impairment	Mild cognitive impairment	Moderate cognitive impairment	Mild cognitive impairment
Apraxia severity	NA	Mild apraxia of speech	Mild apraxia of speech	None	Moderate apraxia of speech	Mild–moderate apraxia of speech
Type of stroke	NA	Hemorrhage	Hemorrhage	Hemorrhage	Hemorrhage	Hemorrhage
Localization	NA	Left frontal lobe	Left frontal lobe, Middle and inferior frontal gyri	Left frontal and parietal lobes	Left posterior lateral temporal lobe with small supratentorial deep watershed infarcts	Left insula extending to the left frontal operculum and left parietal lobe

*Note*: Language Experience and Proficiency Questionnaire control group and individual (bilingual persons with aphasia) data. Self-rated proficiency is reported on a scale from 0 (*none*) to 10 (*perfect*) with the midpoint (5) reflecting *adequate* proficiency. On the language dominance composite score, a negative value means English dominance; a positive value means Spanish dominance. NA = not applicable. DNT = did not test.

#### 2.1.1. Age of language acquisition and proficiency

Eleven control participants and four of the five bilinguals with aphasia acquired Spanish first (L1) followed by English (L2). Ten control participants and one bilingual with aphasia (PT7) acquired English first (L1) followed by Spanish (L2). Age of English acquisition ranged from 0–34 (*mean age* = 11.5 years, *SD* = 8.8) in the controls and 0–16 (*mean age* = 7.8 years, *SD* = 6) in the participants with aphasia, suggesting simultaneous to late sequential bilingualism across the sample. Age of Spanish acquisition ranged from 0–7 (*mean age =* 1*year, SD =* 1.8) in all controls and 0–5 (*mean age* = 1.6 years, *SD* = 2) in the participants with aphasia. PT5 and PT7 were early bilinguals who acquired both languages simultaneously (between ages 0–5 years old) while PT1, PT4 and PT9 acquired English (L2) during their school years (between ages 7–16 years old).

As expected, bilinguals with aphasia reported greater pre- than post-stroke proficiencies across both of their languages. Self-rated proficiency was averaged across speaking, reading and understanding in each language on a scale of 0 (*none*)–10 (*perfect*). Self-rated English proficiency of the control group ranged from 4–10 (*mean* = 8.2, *SD* = 1.7) and was not significantly different, *t*(23) = 1.7, *p* = .089, from the pre-stroke self-reported English proficiency of bilinguals with aphasia (*range* = 9–10, *mean* = 9.8, *SD* = 2.5). Self-reported Spanish proficiency in controls matched pre-stroke Spanish proficiency in bilinguals with aphasia (controls: *range = 5.7–10, mean* = 8.7, *SD = 1.5*; bilinguals with aphasia: *range: 8–10, mean =* 8.9*, SD =* 2.3; *t*(23) = .42, *p* = .671). Post-stroke English proficiency of bilinguals with aphasia ranged from 3.7–9 (*mean* = 5.7, *SD* = 2.5) and post-stroke Spanish proficiency ranged from 2.7–8 (*mean* = 6.3, *SD* = 2.1). Finally, three of five bilinguals with aphasia, as well as the control group, reported greater current exposure to English than Spanish.

#### 2.1.2. Neuropsychological assessments

Testing was conducted in Spanish and English. Both groups were administered the following assessments: *Montreal Cognitive Assessment* (MOCA; Nasreddine et al., 2005), subtests of the *Cognitive Linguistic Quick Test* (CLQT; Helm-Estabrooks, 2002) and the Multilingual Naming Test (MINT; Gollan et al., 2012, see Table 2). On average, cognitive scores fell within the normal range based on published norms and contributed to providing a baseline of cognitive function in the older adult controls. The performance of bilingual persons with aphasia on the CLQT varied from none to moderate impairment.

**Table 2.** Cognitive assessments: Montreal Cognitive Assessment (MOCA); Cognitive Linguistic Quick Test, symbol cancelation (CVLT-SC); Cognitive Linguistic Quick Test, design memory (CVLT-DM) Table 2. Long description.

	Controls		Bilinguals with aphasia (n = 5)				
	Max. scores	Mean (SD; range)	PT1	PT4	PT5	PT7	PT9
**MOCA**	30	.87 (.09; .66–1)	.53	.47	.67	.82	.67
**CLQT-SC**	12	.99 (.03; .91–1)	DNT	0	1.0	1.0	1.0
**CLQT-DM**	6	.87 (.11; .66–1)	DNT	.83	.67	1.0	.67

*Note*: Max scores are reported as raw scores and patient performance is reported as a proportion correct of this raw score. The MOCA was administered in participants’ more dominant language. DNT = did not test.

In addition, bilingual persons with aphasia completed both Spanish and English versions of the Apraxia Battery for Adults-Second Edition (ABA-2; Dabul, 2000) and Spanish and American English versions of the Bilingual Aphasia Test (BAT; Paradis, 2004). Subtests from BAT Parts B and C were incorporated (see Table 3). Subtests from the BAT part C assessing crosslinguistic strengths include word recognition of translation equivalents and translation into the assessed language. Participants were able to accurately identify translation equivalents and produce translations across languages, with stronger performance when translating from Spanish to English than vice versa. Participants’ linguistic impairments ranged from mild to moderate expressive and receptive aphasia, with comorbid mild to moderate cognitive impairment.

**Table 3.** Assessments Table 3. Long description.

	Max. scores/impairment thresholds	PT1		PT4		PT5		PT7		PT9	
		ENG	SPN	ENG	SPN	ENG	SPN	ENG	SPN	ENG	SPN
**MINT**	68	.64	.71	.78	.31	.78	.75	.47	.19	.86	.81
**BAT-B:** simple and semi-complex demands	5	1.0	.8	1.0	1.0	1.0	1.0	1.0	1.0	1.0	1.0
**BAT-B:** verbal auditory discrimination	18	.78	1.0	.95	.83	.77	.95	.89	.72	.83	1.0
**BAT-B:** semantic categories	5	1.0	1.0	1.0	1.0	1.0	.75	.8	.4	1.0	1.0
**BAT-B:** repetition of words, nonsense words and lexical decision	60	.92	.98	1.0	.9	.83	.97	.87	.78	.89	.96
**BAT-B:** verbal fluency	NA	6	10	16	9	30	21	13	4	12	12
**BAT-B:** listening comprehension	5	.8	.6	.8	1.0	1.0	.8	1.0	0	.8	1.0
**BAT-B:** reading comprehension for words	10	1.0	.8	1.0	.8	1.0	.9	1.0	.8	1.0	1.0
**BAT-B:** reading comprehension for sentences	10	.6	.8	.9	.7	.9	.9	.8	.4	.8	1.0
**BAT-C:** word recognition^a^	1.0	1.0	1.0	1.0	1.0	1.0	1.0	1.0	1.0	1.0	1.0
**BAT-C:** translation of words^a^	10	.8	.9	.5	.7	1.0	1.0	.2	.3	.6	.8
**ABA–2:** diadochokinetic rate	26+ NI 7–25 MI	31	19	29	22	21	32	19	9	7	19
**ABA–2:** increasing word length (a)	<1 NI 2–4 MI 5–7 MO 8+ SI	2	0	0	0	−1	0	2	16	5	5
**ABA–2:** increasing word length (b)	<1 NI 2 MI 3–5 MO 6+ SI	3	0	0	−4	2	0	6	DNT	11	6
**ABA–2:** limb apraxia	44–50 NI	50	44	45	45	50	45	50	41	50	50
**ABA–2:** oral apraxia	44–50 NI	50	45	50	50	45	50	50	50	50	48
**ABA–2:** latency time and utterance time for polysyllabic words	0–15 NI 16–55 MI	21	9	14	47	5	20	42	56	27	32
**ABA–2:** repeated trials	28–30 NI 16–27 MI 5–15 MO	26	30	30	27	30	30	25	7	26	23
**ABA–2:** inventory of articulation characteristics of apraxia	NA	8	5	0	4	7	5	9	12	6	5

*Note*: Spanish and English Multilingual Naming Test (MINT), Bilingual Aphasia Test (BAT) and the Apraxia Battery for Adults (ABA-2) diagnostic scores for bilingual persons with aphasia. BAT VF has no maximum score. Max scores are reported as raw scores and patient performance is reported as a proportion correct of this raw score. DNT = did not test; MI = mild impairment; MO = moderate impairment; NA = not applicable; NI = no impairment; SI = severe impairment.

a For BAT part C translation tasks, findings are reported in terms of the source language of translation; for example, Spanish-to-English translations are reported in the Spanish column.

#### 2.1.3. Language dominance profiles

Language dominance profiles were determined by combining variables that were theoretically related and numerically correlated based on previous studies (see Gálvez-McDonough et al., 2025; Robinson Anthony & Blumenfeld, 2019) into a composite score for each participant. This included self-rated proficiency speaking, reading and understanding in each language (English: *rs* = .79–.84; Spanish: *rs* = .80–.91), as well as self-reported proficiency and exposure in each language (English: *rs* = .22–.34; Spanish: *rs* = .33–.47). This yielded a continuous variable ranging from negative to positive values between −1 and 1. Negative composite scores indexed English language dominance while positive composite scores indexed Spanish dominance. A value of 0 indexed a balanced language dominance profile. Language dominance scores are included in Table 1.

For controls, language dominance profiles were comparably distributed across both languages. In general, participants showed slightly unbalanced language profiles, and this tended slightly toward larger English versus Spanish dominance (mean composite score = − .065). According to the post-stroke language dominance composite score, in the bilingual persons with aphasia, three individuals were considered more English dominant, one more Spanish dominant and one had a more balanced bilingualism profile.

#### 2.1.4. MRI lesion mapping in bilinguals with aphasia

**Figure 1.**
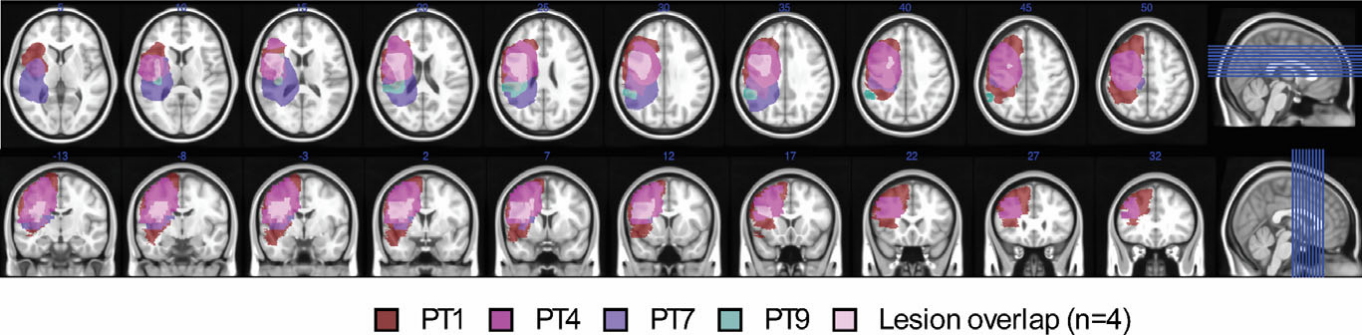
Lesion overlap of four bilingual participants with aphasia. Each participant is color coordinated to visualize the size of their respective lesion in the left hemisphere. The light pink color represents the regions impaired across all participants. Figure 1. Long description.

Magnetic resonance imaging scans were retrieved from 4 of the 5 bilinguals with aphasia to confirm a left hemisphere lesion distribution and disruption of brain regions subserved by the left middle cerebral artery territory (see Figure 1). Each participant’s lesion was manually segmented in ITK-SNAP (Yushkevich et al., 2016), and both the lesion and the MRI scans were transformed to MNI space using FMRIB Software Library (FSL; Jenkinson et al., 2012). A lesion overlay was generated to show the percentage of voxel lesion overlap across participants using MRIcron (Rorden, 2025). To determine the extent of lesion damage, we computed the overlap between each participant’s lesion mask and anatomical regions defined by the Automated Anatomical Labeling atlas (AAL; Tzourio-Mazoyer et al., 2002) implemented in MRIcroGL (Brett et al., 2001). For each region of interest, we extracted the absolute number of lesioned voxels and calculated the percentage of voxel-wise damage relative to the total voxel count within that region (Table 4). Overall, the lesion distribution underscores the widespread impact of left-hemisphere damage across regions supporting both the bilingual language control network (Abutalebi & Green, 2007, 2013, 2016) and cognitive control (Calabria et al., 2018).

**Table 4.** Lesioned brain regions and voxel damage Table 4. Long description.

Anatomical classification	Regions	All	PT1	PT4	PT7	PT9	
**Frontal**	***Left precentral gyrus***	93%	92%	85%	13%	17%	
**Frontal**	***Left supplemental motor area* *(SMA)***	50%	47%	15%	<1%	-	
**Prefrontal**	***Left anterior cingulate cortex* *(ACC)***	<1%	-	<1%	-	-	
**Prefrontal**	***Left superior frontal gyrus* *(SFG)***	45%	45%	21%	-	-	
**Prefrontal**	***Left middle frontal gyrus* *(MFG)***	52%	47%	40%	<1%	1%	
**Prefrontal**	***Left inferior frontal gyrus, pars opercularis* *(IFG)***	90%	89%	68%	16%	39%	
**Prefrontal**	***Left inferior frontal gyrus, pars triangularis* *(IFG)***	47%	38%	28%	<1%	5%	
**Parietal**	***Left inferior parietal cortex***	75%	67%	5%	12%	8%	
**Subcortical**	***Left insula***	82%	81%	26%	42%	21%	
**Subcortical**	***Left caudate***	20%	<1%	18%	7%	-	
**Subcortical**	***Left putamen***	55%	49%	6%	40%	<1%	
**Subcortical**	***Left thalamus***	21%	1%	-	20%	-	
Frontal	*Left dorsal cingulate cortex* *(dACC)*	30%	5%	23%	9%	-	
Frontal	Left rolandic operculum	91%	82%	66%	71%	31%	
Parietal	*Left angular gyrus*	54%	4%	-	51%	5%	
Parietal	*Left supramarginal gyrus*	71%	41%	1%	57%	30%	
Parietal	Left superior parietal	20%	20%	<1%	-	-	
Temporal	Left Heschl’s gyrus	99%	56%	45%	93%	32%	
Temporal	*Left middle temporal gyrus* *(MTG)*	25%	5%	-	20%	<1%	
Temporal	*Left superior temporal gyrus* *(STG)*	68%	24%	5%	48%	9%	
Subcortical	*Left pallidum*	20%	20%	-	4%	-	

*Note*: Percentage of voxel damage identified through a region of interest approach. Italicized regions correspond to regions associated with language-related functions (Calabria et al., 2018; Price, 2012). Bolded regions align with regions in the bilingual language control network (Green & Abutalebi, 2013); non-bolded regions represent additional areas with >20% voxel damage in the lesion overlay. Cells marked “-” indicate no voxel damage was observed in a region for a specific participant (see Figure 1).

### 2.2. Study design

Participants performed an expressive crosslinguistic version of the picture word interference task (PWI, e.g., Rosinski et al., 1975; Lupker, 1979) and a nonlinguistic arrow version of the Stroop task (e.g., Giezen et al., 2015). These two tasks are often implemented to investigate attentional control processes in the linguistic (e.g., Capizzi et al., 2017; Lupker, 1979; Roelofs, 2003) and nonlinguistic domains (e.g., Blumenfeld & Marian, 2014; Lehtonen et al., 2018; Zhou & Krott, 2016). The current study followed a 3 × 2 × 2 design, with trial type (incongruent, congruent, neutral) and task (linguistic PWI task, nonlinguistic spatial Stroop task), as within-subject factors, and group (healthy controls, bilinguals with aphasia) as a between-subjects factor.

#### 2.2.1. Expressive picture-word interference task

To examine crosslinguistic competition during naming, a PWI task was employed where participants named a picture in Spanish while ignoring a superimposed word presented at the same time as the picture. Stimuli consisted of 42 photographs of common objects selected through Google image searches (only images with Creative Commons licenses were selected). Stimuli from the Bank of Standardized Stimuli (BOSS; Brodeur et al., 2010) were also included. Stimuli were centered against a white, square background and superimposed with a Spanish distractor word that was written in all-capital black Arial size 26 font over the center of the image (500 × 500 pixels) with a visual angle of 3° 47′ 0.32″. Name agreements on all images were collected on an unrelated sample of 10 participants. All stimuli were associated with name agreements of 80% or higher.

Critical stimuli were repeated across three conditions: a False Cognate condition (FC), an Unrelated (UR) condition and an Identity (ID) condition (see Figure A1 and Table A3 in the Supplementary Materials). In the crosslinguistic FC condition, the distractor word was similar in phonological and orthographic form but different in meaning compared to the English picture name (e.g., picture – *uva* or “grape”; superimposed word – GRAPA, meaning “staple” in English but close to “grape” in form). In the UR condition, the picture and the word did not match in either form or meaning (i.e., picture – *uva* or “grape”; word – OLA English “wave”). We measured the phonological overlap between false cognate pairs and unrelated pairs using the Crosslinguistic Overlap Scale for Phonology (COSP; Kohnert et al., 2004). As planned, the COSP scores were significantly higher for false cognate pairs than for unrelated pairs in our stimuli, *t*(20) −10.97, *p* < .001; mean COSP score for UR pairs: 1.81, SD: .93, range = 0–3; mean COSP score for FC pairs: 6, SD: 1.52, range = 2–8. All of the superimposed distractor words were represented as images in other trials, meaning that the distractor words were a part of the response set. This design was chosen to maximize interference, as shown in the semantically related version of the PWI paradigm (Piai et al., 2012).

The PWI task consisted of 420 trials (84 FC, 168 UR and 168 ID trials) that were equally distributed across 6 blocks of 70 trials each. The order of stimulus presentation was mixed pseudorandomly across conditions using Mix (van Casteren & Davis, 2006), with a minimum distance of five different pictures presented between repetitions of both stimulus and distractor items and a maximum of two trials in a row in the same condition. Additional stimulus sequencing constraints included: no more than one repetition in a row of stimuli belonging to the same semantic category, at least three trials between stimuli that started with the same first phoneme and stimuli with the same grammatical gender in Spanish repeated no more than three times in a row. To further avoid order effects, we created six different lists with separate stimulus sequences, each used at least 3 times across participants.

Word length ranged from three to 11 characters in Spanish and three to 10 characters in English. Syllable length ranged from one to four syllables in both languages. Lexical frequency of stimuli ranged from .17 to 137.98 words per million in Spanish (*mean =* 20.72, *SD =* 31) and .75 to 130.61 words per million in English (*mean =* 26.61, *SD =* 30.5), as derived from the SUBTLEX-ESP database (Cuetos et al., 2011). A difference in lexical frequency between the two languages was present when naming pictures, *t*(102) = −2.2, *p* = .033, and viewing words, *t*(102) = −2.03, *p* = .045. The higher word frequency in English than in Spanish is expected to enhance the crosslinguistic interference effect and to affect all conditions equally. Grammatical gender of words was balanced across conditions to control for potential gender congruency effects (Sá-Leite et al., 2022). The proportion of grammatical gender congruency between the picture and the word was matched in FC and UR trials.

Each trial consisted of the following: (1) a .3–.7 sec jittered fixation cross was displayed for 1000 ms at the center of the screen, (2) the stimulus picture overlaid with a word for 3000 ms and (3) a white blank screen for 2000 ms. The word that was overlaid belonged to one of the three tested conditions. Participants were instructed to name the picture in Spanish as quickly and as accurately as possible.

#### 2.2.2. Nonlinguistic spatial Stroop task

An arrow version of the Stroop task (also referred to as the spatial Stroop Task and the spatial Simon Task; Hilchey & Klein, 2011) was employed to examine participants’ nonlinguistic cognitive control abilities. Stimuli consisted of left- or right-pointing arrows in white on a black background (with a visual angle of 3° 49′ 0.10″). Three experimental conditions were tested: congruent, incongruent and neutral stimuli. On congruent trials, right- and left-pointing arrows appeared on the right and left sides of a centered fixation cross, matching location and direction. On incongruent trials, arrow direction and location were mismatched, generating stimulus-internal conflict. On neutral trials, stimuli appeared centrally instead of the fixation cross. Participants indicated arrow direction by pressing “3” with their right hand for right-pointing arrows and “1” with their left hand for left-pointing arrows. To avoid list effects, one of three randomized lists was presented to each participant, and a similar number of participants saw each list.

The trial sequence is depicted in the supplementary materials and consisted of the following: (1) a .3–.7 sec jittered fixation cross was displayed for 1000 ms at the center of the screen; (2) the stimulus picture for 700 ms; (3) a black blank screen for 2000 ms. Stimuli were presented across eight blocks, with 55 trials per block, adding up to a total of 440 trials (264 congruent, 88 incongruent and 88 neutral trials). Within each condition, there was an equal percentage of left- and right-pointing arrows (see Figure A2 in the supplementary materials). Incongruent trials made up 20% of all trials in each task. This percentage was chosen in an effort to minimize proactive awareness of conflict trials in order to maximize interference effects, as in previous studies (Blumenfeld & Marian, 2013; Botvinick et al., 2001).

### 2.3. Procedure

The study was conducted over the course of three to five sessions to reduce fatigue effects. After giving consent and completing background and neuropsychological assessment, individuals participated in two EEG tasks: the crosslinguistic PWI task followed by the nonlinguistic spatial Stroop task.

Participants were seated approximately 150 cm away from the stimulus monitor in a sound-attenuated, dimly lit chamber separate from the experimenter. The presentation monitor was situated on the other side of a glass window outside of the chamber to reduce possible electrical noise in the EEG recordings. Reaction times and accuracy rates were recorded using a microphone. Both the linguistic and nonlinguistic tasks were coded and presented on Presentation software (version 18.0, Neurobehavioral Systems, Inc., Berkeley, CA, www.neurobs.com).

Participants first completed the PWI task, followed by the nonlinguistic spatial Stroop task. At the beginning of each task, participants were presented with a short familiarization block. Participants named picture targets without their respective articles (e.g., *ciruela*, not *la* ciruela). Reaction times for each trial were measured as the difference between the time of stimulus presentation and the time of vocal onset or button press, with a timeout value set to 3000 ms. Breaks between trial blocks were self-paced across tasks.

Electroencephalography was recorded using an elastic electrode cap with 64 Ag/AgCl active electrodes (10–20 system positions). The vertical electrooculogram (EOG) was recorded using one surface electrode placed below the left eye and the electrode above the left eye, at the Fp1 recording site. The passive references were placed over the left and right mastoids. EEG was amplified with an ActiCHamp bio-amplifier with a bandpass of DC to 100 Hz (3 db/octave) and was sampled continuously at 250 Hz with acquisition filters.

Following EEG, participants were administered the neuropsychological tasks in English that they had previously completed in Spanish. Task order was strategically designed to ensure that participants completed Spanish tasks prior to starting the Spanish experimental PWI task. Spanish neuropsychological tasks were administered in session 1, and English tasks in session 3 (controls) or in sessions 4–5 (bilinguals with aphasia), allowing maximum time to elapse between the Spanish and English versions. The spatial Stroop task was also always performed after the PWI task to further separate the Spanish- and English-speaking portions of the study.

### 2.4. Analysis

#### 2.4.1. Data pre-processing

During *behavioral data pre-processing*, response accuracy and verbal reaction times were measured using the software CheckVocal (Protopapas, 2007). Trials were coded as errors when participants produced the distractor word instead of the image name, named the image in English instead of Spanish, or produced a phonetically different word with a different semantic meaning. Non-target responses were considered correct if they were consistent synonyms of the stimulus image (i.e., *pizarron* instead of target *pizarra* or *elote* instead of target *maiz*). Although these words are interchangeable in Spanish, these trials were removed from the analysis given that they were not the targeted crosslinguistic false cognate (accounting for less than 4% of trials). Trials were excluded from analysis when participants produced any kind of verbal (e.g., complete or partial) or button-press error, when the participant did not answer within the 3000 ms limit or when a switch to the non-target language was made (less than 1% of trials).

*EEG and EMG* data were recorded using Brain Vision Analyzer (BrainVision Analyzer, version 2.2.0; Brain Products GmbH, Gilching, Germany). Ocular artifacts were removed using independent component analysis as implemented in EEGLAB (Delorme & Makeig, 2004). A blind source separation algorithm based on canonical correlation analysis (BSS-CCA; De Clercq et al., 2006) using the AAR toolbox in EEGlab (Gomez-Herrero et al., 2006) was applied twice: first on non-overlapping consecutive 30 sec time windows to reduce tonic EMG activity from frowning and/or muscle fatigue, and second on non-overlapping 2 second-long time windows to target EMG activity from articulation (Anderson et al., 2022; Mendoza et al., 2021; Riès et al., 2020; 2021; 2011; 2013; 2015; Vos et al., 2010). Any artifacts remaining after BSS-CCA were manually rejected on a trial-by-trial basis. In addition, Laplacian transformation is known for its sensitivity to local artifacts (i.e., artifacts present at single electrodes: phase artifacts as well as slow electrical shifts), which were also removed. A total of three channels were removed (i.e., F7, C3, CP3) in the PWI task and two channels (i.e., C3 and Pz) from the spatial Stroop due to poor signal quality in at least one of the included participants at these recording sites. Importantly, none of these channels were direct neighbors of our electrode of interest (FCz), and their removal therefore did not affect the estimation of the current source density at this site. Both the stimulus and the response-locked averages were baseline-corrected using the −500 to stimulus onset time window. Laplacian transformation (i.e., current source density estimation), as implemented in Brain Vision Analyzer (Brain Products, Munich, Legendre polynomial: 15° maximum), was then applied for each participant as in previous studies (Anderson et al., 2022; Mendoza et al., 2021; Riès et al., 2020; 2021; Riès et al., 2011, 2013; 2015). This method provides an estimate of the local current source density at each electrode site (Nunez et al., 1994). We chose three for the degree of spline because this value best minimizes errors (Perrin et al., 1987). We assumed a radius of 10 cm for the sphere representing the head. The resulting unit was μV/cm^2^. The enhanced topographical localization from Laplacian transformation allowed us to focus our analysis on a medial frontal site known to be associated with the processes of interest during language production (Mendoza et al., 2021; Riès et al., 2021; Riès et al., 2011, 2013; Vidal et al., 2000).

#### 2.4.2. Data processing

Statistical analyses were performed within R version 4.3.1. During *behavioral data processing*, generalized linear and logistic mixed effect models were computed to analyze the effects of the independent variables of interest on reaction times and accuracy rates, respectively (Baayen et al., 2008; Jaeger, 2008). For both tasks, the individual reaction times were inversed to reduce the skewed distribution of RTs and approach a normal distribution. We tested for fixed effects of Condition (False cognate versus Unrelated and Identity versus Unrelated in the PWI task, and Incongruent versus Neutral and Congruent versus Neutral in the spatial Stroop task) and Group (bilingual persons with aphasia versus controls) and controlled for random effects (picture name, stimulus direction and participant), as well as by-item and by-participant random slopes for Condition. A restricted maximum likelihood model (REML) was implemented to determine model fit for reaction times and t-tests using Satterthwaite’s method. While a maximum likelihood was used to determine model fit for accuracy rates with the control BOBQA optimizer, the *p* values were obtained by using the package lme4 (Bates et al., 2015).

During *ERP data processing*, EEG data analysis was performed at electrode site FCz based on our a-priori hypotheses and previous studies (e.g., Mendoza et al., 2021; Riès et al., 2013; Vidal et al., 2011). Statistical analysis was conducted on Laplacian-transformed stimulus-locked and response-locked averages using three types of measures: (1) the slopes of the activity within the windows of interest, (2) the peak-to-peak amplitude (i.e., the difference in amplitude between two consecutive peaks of activity with opposite polarities) and (3) the latency of the peaks of interest. Slopes were measured by fitting a linear regression to the group and individual participant data to substantiate the existence of a component by comparing the data to a norm (zero) within a given window of interest (e.g., Riès et al., 2011; 2013). Analysis was performed using two-tailed student t tests and ANOVAs for comparisons of more than two groups of means. The peak-to-peak amplitudes were measured as described in Riès et al. (2013). Finally, the latencies of these peaks were measured on smoothed data to reduce the impact of background noise.

## 3. Results

### 3.1. Linguistic results

#### 3.1.1. Behavioral results – PWI task

**Figure 2.**
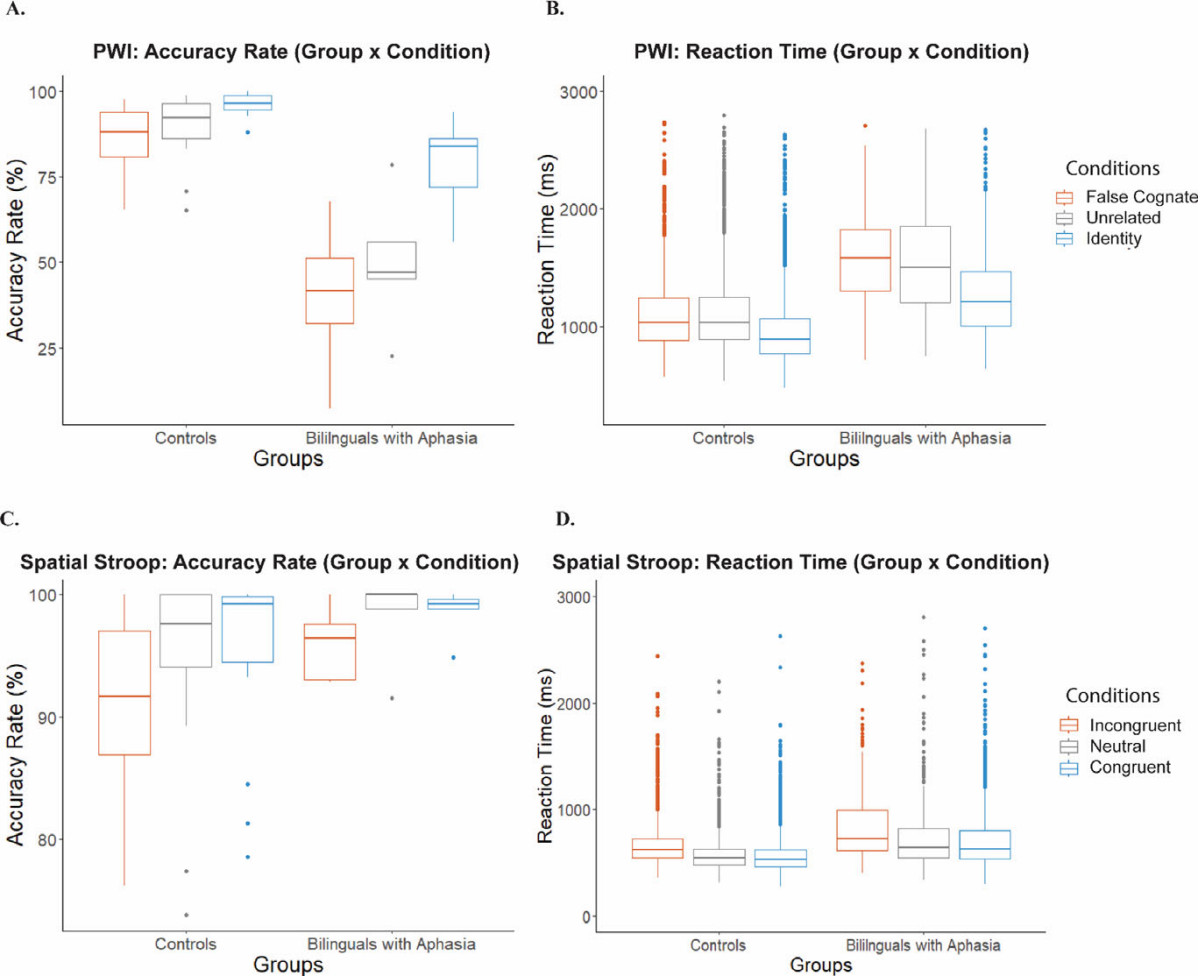
A) Accuracy rates between controls and bilinguals with aphasia across mean conditions of FC, ID and UR in the linguistic PWI task. B) Reaction times between controls and bilinguals with aphasia across false cognate, identity and unrelated conditions in the PWI task. C) Accuracy rates between controls and bilinguals with aphasia across conditions in the nonlinguistic spatial Stroop task. D) Reaction times between controls and bilinguals with aphasia across incongruent, congruent and neutral conditions in the spatial Stroop task. *Note:* The horizontal lines indicate the median accuracy and reaction times per condition. Figure 2. Long description.

The mean accuracy rates, reaction times, standard deviations and ranges are presented in each task across groups in Table 5. There were significant main effects of Condition (*Χ* ^2^ (2,24) = 33.04, *p* < .001) and Group (*Χ* ^2^ (1,24) = 31.60, *p* < .001) on Accuracy (see Figure 2A). Bilinguals with aphasia were significantly less accurate at naming pictures than controls (*β*_raw_ = −2.747, *SE* = .489, Wald *Z* = −5.62, *p* < .001). There was a significant crosslinguistic interference effect, as indexed by lower accuracy on false cognate versus unrelated trials (*β*_raw_ = −.435, *SE* = .105, Wald *Z* = −4.16, *p* < .001), and a significant within-language facilitation effect, as indexed by higher accuracy on identity versus unrelated trials (*β*_raw_ = .729, *SE* = .128, Wald *Z* = 5.70, *p* < .001). In addition, there was a marginal interaction between Condition and Group on Accuracy (*Χ* ^2^ [2,24] = 5.68, *p* = .058): Bilinguals with aphasia had a significantly greater within-language facilitation effect, as indexed by higher accuracy on identity versus unrelated trials, compared to controls (*β*_raw_ = .470, *SE* = .197, Wald *Z* = 2.38, *p* = .017). There was no significant interaction between Group and the crosslinguistic interference effect on Accuracy (*β*_raw_ = −.198, *SE* = .132, Wald *Z* = −1.49, *p* = .134).

**Table 5.** Behavioral data Table 5. Long description.

	PWI		Spatial Stroop	
	Mean accuracy rate (SD; range)	Mean reaction time (SD; range)	Mean accuracy rate (SD; range)	Mean reaction time (SD; range)
Controls (n = 21)	91.79% (6.49%; 80–99%)	1047 ms (323 ms; 774–1321 ms)	94.62% (6.6%; 77–100%)	585 ms (180 ms; 277–2624 ms)
Bilinguals with aphasia (n = 5)	59.33% (18%; 33–80%)	1419 ms (435 ms; 1218–1681 ms)	97.91% (2.36%; 94–100%)	755 ms (328 ms; 298–2804 ms)
PT1	70.24%	1470 ms	99.76%	621 ms
PT4	54.05%	1681 ms	98.10%	783 ms
PT5	79.52%	1218 ms	99.29%	543 ms
PT7	32.86%	1366 ms	93.84%	621 ms
PT9	60.00%	1419 ms	98.56%	1188 ms

*Note*: Mean accuracy rate and reaction time per task across adult controls and bilinguals with aphasia (individual data included).

There were also significant main effects of Condition (*Χ* ^2^ (2,24) = 96.27, *p* < .001) and Group (*Χ* ^2^ (1,24) = 25.69, *p* < .001) on reaction times (RTs) (see Figure 2B). Bilinguals with aphasia were significantly slower at naming pictures compared to controls (*β*_raw_ = −3.106x10^−04^, *SE* = 6.129x10^−05^, *t* = −5.07, *p* < .001). Significant crosslinguistic interference (*β*_raw_ = −4.863 x10^−05^, *SE* = 8.033x10^−06^, *t* = −6.053, *p* < .001) and within-language facilitation (*β*_raw_ = 1.071x10^−04^, *SE* = 1.162x10^−05^, *t* = 9.218, *p* < .001) effects were also present on RT. There was no significant interaction effect between Group and Condition on RT (*Χ* ^2^ (2,24) = 2.23, *p* = .327).

We report individual participant data for bilinguals with aphasia in Table 5. All bilinguals with aphasia were the least accurate in the false cognate condition compared to identity and unrelated conditions (FC accuracy range: 7–68%; ID accuracy range: 56–94%, UR accuracy range: 23–79%). For RTs, all bilinguals with aphasia were quickest in identity trials (ID range: 1128-1505 ms) and the slowest in false cognate trials (FC range: 712-2797 ms) compared to unrelated trials (UR range: 607-2900 ms), except for PT1 who was slightly slower in unrelated trials compared to false cognate trials (1662 ms versus 1652 ms).

#### 3.1.2. PWI task stimulus-locked electrophysiological results

**Figure 3.**
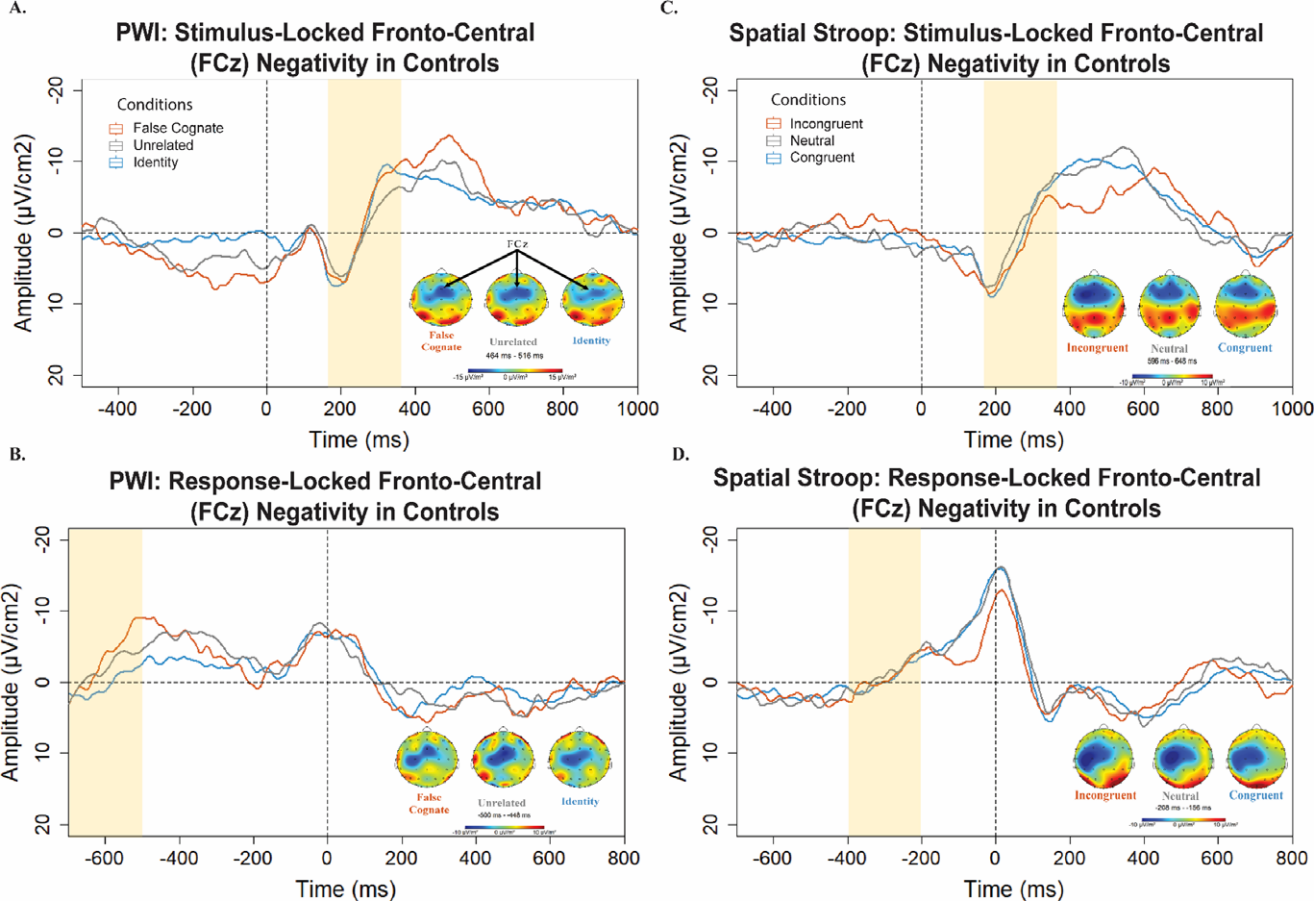
A) Adult control *stimulus*-locked Laplacian-transformed ERP waveforms during the *PWI* task at electrode FCz, pictured on the scalp across conditions. Topographies illustrate the scalp distribution of electrical activity during the window centered on the peak response in the interference condition (i.e., False cognates and Incongruent). B) Adult control *response*-locked Laplacian-transformed ERP waveforms during the *PWI* task at electrode FCz. C) Adult control *stimulus*-locked Laplacian-transformed ERP waveforms during the *spatial Stroop* task at electrode FCz. D) Adult control *response*-locked Laplacian-transformed ERP waveforms during the *spatial Stroop* task at electrode FCz. *Note:* Negative amplitude is plotted up in this diagram. Figure 3. Long description.

In the control group, the slope of the negative-rising component observed at FCz was significantly different from zero between 180 and 380 ms post-stimulus onset overall, *t*(19) = −6.97, *p* < .001, and within each conditions (FC: *t*(19) = −6.89, *p* < .001; ID: *t*(19) = −6.21, *p* < .001; UR: *t*(19) = −4.98, *p* < .001). The slopes were not significantly different between conditions, *F*(2,57) = .898, *p* = .413; multiple comparisons test using the Tukey method: FC versus UR: *p*_adj_ = .409, ID versus UR: *p*_adj_ = .597. This negativity peaked on average at 424 ms (*SD = 98* ms) after stimulus presentation across conditions. No significant difference on the peak-to-peak amplitude was detected between false cognates and unrelated conditions, *t*(19) = −1.27, *p* = .218, or between the identity and unrelated conditions, *t*(19) = −.54, *p* = .592. The latencies were also not significantly different between conditions, *F*(2,57) = 1.008, *p* = .371; multiple comparisons test using the Tukey method: FC versus UR: *p*_adj_ = .455, ID versus UR: *p*_adj_ = .428; see Figure 3A.

**Figure 4.**
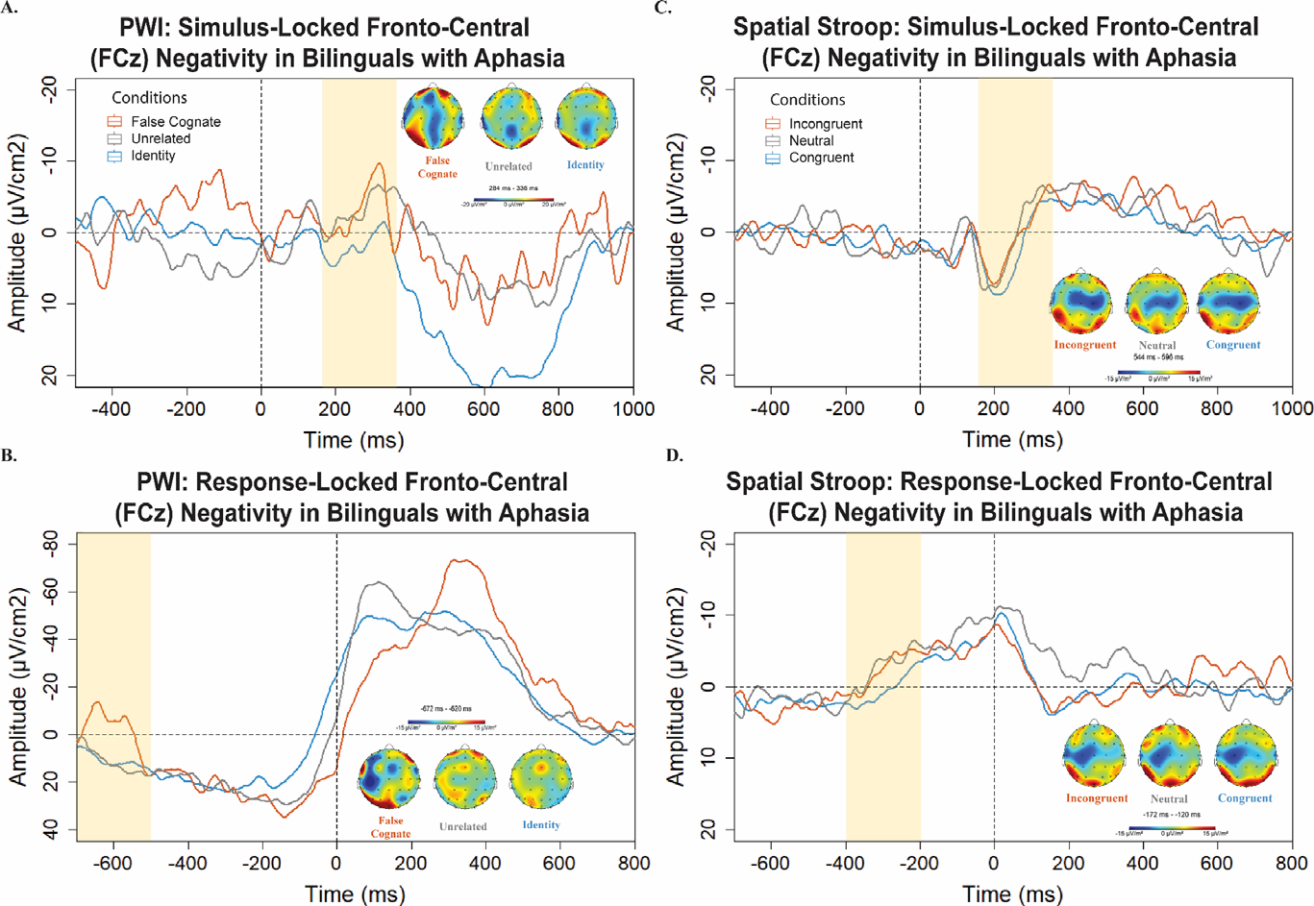
A) Bilinguals with aphasia *stimulus*-locked Laplacian-transformed ERP waveforms during the *PWI* task at electrode FCz, pictured on the scalp across conditions. Topographies illustrate the scalp distribution of electrical activity during the window centered on the peak response in the interference condition (i.e., False cognates and Incongruent). B) Bilinguals with aphasia *response*-locked Laplacian-transformed ERP waveforms during the *PWI* task at electrode FCz. C) Bilinguals with aphasia *stimulus*-locked Laplacian-transformed ERP waveforms during the *spatial Stroop* task at electrode FCz. D) Bilinguals with aphasia *response*-locked Laplacian-transformed ERP waveforms during the *spatial Stroop* task at electrode FCz. *Note:* Negative amplitude is plotted up in this diagram. Figure 4. Long description.

For Bilinguals with aphasia, the slope of the ERP activity was not significantly different from zero between 180 and 380 ms overall, *t*(4) = −1.08, *p* = .339. The slopes were not significantly different between conditions, *F*(2,12) = .821, *p* = .463; multiple comparisons test using the Tukey method: FC versus UR: *p*_adj_ = .436, ID versus UR: *p*_adj_ = .732; see Figure 4A.

#### 3.1.3. PWI task response-locked electrophysiological results

In controls, the slope of the medial-frontal negativity at FCz between −700 to −450 ms pre-vocal onset differed significantly from zero overall, *t*(19) = −6.39, *p* < .001, and within each condition (FC: *t*(19) = −6.61, *p* < .001; ID: *t*(19) = −2.85, *p* = .1; UR: *t*(19) = −2.78, *p* = .01). The slopes were not significantly different between conditions, *F*(2,57) = 1.753, *p* = .182; multiple comparisons test using the Tukey method: FC versus UR: *p*_adj_ = .450, ID versus UR: *p*_adj_ = .804. The peak-to-peak amplitude was marginally larger in false cognates compared to unrelated conditions, *t*(19) = −1.96, *p* = .065. No significant difference in peak-to-peak amplitude was detected between identity and unrelated conditions, *t*(19) = .65, *p* = .523. The negativity peaked on average at -521 ms (*SD =* 116 ms) before the response across conditions. The latencies were not significantly different between conditions, *F*(2,57) = .224, *p* = .8; multiple comparisons test using the Tukey method: FC versus UR: *p*_adj_ = .784, ID versus UR: *p*_adj_ = .961; see Figure 3B.

The same window between −700 and −450 ms was analyzed in bilinguals with aphasia. The slope of the ERP was not significantly different from zero, *t*(4) = .33, *p* = .757. An additional window between −200 and 100 ms was explored given the large negative peak seen on the grand average peaking within 200 ms post-vocal onset; see Figure 4B. The slope of this activity did not differ significantly from zero, *t*(4) = −1.09, *p* = .335.

### 3.2. Nonlinguistic results

#### 3.2.1. Behavioral results – spatial Stroop task

There was a significant main effect of Condition (*Χ* ^2^ [2,23] = 29.15, *p* < .001) but not of Group on Accuracy (*Χ* ^2^ [1,23] = 1.03, *p* = .310), indicating that the bilinguals with aphasia were not less accurate as controls in the spatial Stroop task (*β*_raw_ = .754, *SE* = .743, Wald *Z* = 1.01, *p* = .311). There was a significant Stroop interference effect, as indexed by lower accuracy on incongruent versus neutral trials (*β*_raw_ = −1.117, *SE* = .210, Wald *Z* = −5.31, *p* < .001), and a significant Stroop facilitation effect, as indexed by increased accuracy on congruent versus neutral trials (*β*_raw_ = .798, *SE* = .197, Wald *Z* = 4.04, *p* < .001); see Figure 2C. There was no significant interaction between Group and Condition on Accuracy (*Χ* ^2^ [2,23] = .025, *p* = .987).

There was a significant main effect of Condition (*Χ* ^2^ [2,23] = 202.44, *p* < .001) and Group (*Χ* ^2^ [1,23] = 4.592, *p* = .032) on RTs (see Figure 2D). Bilinguals with aphasia were significantly slower at responding compared to controls (*β*_raw_ = −3.014 × 10^−04^, *SE* = 1.407 × 10^−04^, *t* = −2.143, *p* = .044). Significant Stroop interference (i.e., slower RTs in incongruent versus neutral trials, *β*_raw_ = −1.842 × 10^−04^, *SE* = 1.295 × 10^−05^, *t* = −14.23, *p* < .001) and Stroop facilitation effects were observed (i.e., faster RTs in congruent versus neutral trials, *β*_raw_ = 1.228 × 10^−04^, *SE* = 1.021 × 10^−05^, *t* = 12.02, *p* < .001) were present. There was no significant interaction between Group and Condition on RT (*Χ* ^2^ [2,23] = 2.277, *p* = .320).

Individual performance on the spatial Stroop task in bilinguals with aphasia is reported in Table 5. All bilinguals with aphasia were the least accurate in the incongruent condition compared to the congruent and neutral conditions (IC accuracy range: 93–100%; C accuracy range: 95–100%, N accuracy range: 92–100%). All participants with aphasia were the most accurate in the neutral condition compared to the congruent trials, except for PT7, who was most accurate in the congruent condition. For reaction times, all participants were slowest in the incongruent compared to the congruent and neutral conditions (IC reaction time range: 628–1327 ms, C reaction time range: 520–1137 ms, N reaction time range: 531–1256 ms). Most participants, except for PT5 and PT9, were fastest in the neutral condition compared to the congruent condition.

#### 3.2.2. Spatial Stroop task stimulus-locked electrophysiological results

In controls, we observed a medial-frontal negativity at the same recording site as in the linguistic task, FCz, peaking on average 443 ms (*SD =* 160 ms) after stimulus presentation; see Figure 3C. The slope of the negativity was significantly different from zero between 180 and 380 ms overall, *t*(17) = −6.11, *p* < .001, but the slopes were not significantly different between conditions, *F*(2,51) = .662, *p* = .52; multiple comparisons test using the Tukey method: C versus N: *p*_adj_ = .825, IC versus N: *p*_adj_ = .843. The latencies were not significantly different between conditions, (IC: 418 ms*, SD =* 212 ms*;* C: 496 ms, *SD* = 97 ms*;* N: 496 ms, *SD* = 97 ms; *F*(2,51) = 1.332, *p* = .273; multiple comparisons test using the Tukey method: C versus N: *p*_adj_ = .485, ICvN: *p*_adj_ = .906). No significant difference on peak-to-peak amplitude was detected between conditions: incongruent versus neutral conditions, *t*(17) = .98, *p* = .343, congruent versus neutral conditions, *t*(17) = 1.38, *p* = .186. An additional window between 344 and 444 ms was explored to investigate the positive dip seen on the grand average in the incongruent condition; see Figure 3C. The slope of the positive dip in the incongruent condition was not significantly different from the slope of the congruent or neutral conditions (IC versus C: *t*(17) = 1.60, *p* = .128; IC versus B: *t*(17) = .85, *p =* .405).

For bilinguals with aphasia, a smaller negativity was observed peaking on average at 411 ms (*SD =* 222 ms) after stimulus presentation; see Figure 4C. The slope of the negativity was significantly different from zero between 180 and 380 ms across the 5 bilinguals with aphasia, *t*(4) = −4.04, *p* = .016. The slopes were not significantly different between condition, *F*(2,12) =1.229, *p* = .327; multiple comparisons test using the Tukey method: CvsN: *p*_adj_ = .919, IC versus N: *p*_adj_ = .321. The latencies between conditions were not significantly different, *F*(2,12) = .071, *p* = .931; multiple comparisons test using the Tukey method: C versus N: *p*_adj_ = .998, IC versus N: *p*_adj_ = .934. No significant difference on peak-to-peak amplitude was detected across conditions: incongruent and neutral conditions, *t*(4) = .17, *p* = .873, congruent compared to neutral conditions, *t*(4) = −.837, *p* = .449 or incongruent compared to congruent conditions, *t*(4) = 1.05, *p* = .352.

#### 3.2.3. Nonlinguistic task response-locked electrophysiological results

In controls, we observed a medial-frontal negativity at FCz peaking on average −202 ms (*SD* = 47 ms) prior to the response on the grand average; see Figure 3D. The slope of the negativity was significantly different from zero between −400 and −200 ms, *t*(17) = −2.84, *p* = .011. The slopes were not significantly different between conditions, *F*(2,51) = .044, *p* = .957; multiple comparisons test using the Tukey method: C versus N: *p*_adj_ = .954, IC versus N: *p*_adj_ = .979). The latencies were not significantly different between conditions, *F*(2,51) = .841, *p* = .437; multiple comparisons test using the Tukey method: C versus N: *p*_adj_ = .812, IC versus N: *p*_adj_ = .403. The peak-to-peak amplitude of this negativity was significantly larger in neutral compared to congruent conditions, *t*(17) = −3.10, *p* = .006. The peak-to-peak amplitude did not differ significantly between incongruent and neutral conditions, *t*(17) = −.46, *p* = .649.

For Bilinguals with aphasia, a similar negativity was observed peaking on average at −206 ms (*SD* = 16 ms); see Figure 4D. The slope of the negativity was marginally different from zero between −400 and −200 ms across the 5 Bilinguals with aphasia, *t*(4) = −2.47, *p* = .068. The slopes were not significantly different between conditions, *F*(2,51) = .02, *p* = .98; multiple comparisons test using the Tukey method: C versus N: *p*_adj_ = .991, IC versus N: *p*_adj_ = .997. The latencies were not significantly different between conditions, *F*(2,12) = .647, *p* = .541; multiple comparisons test using the Tukey method: C versus N: *p*_adj_ = .711, IC versus N: *p*_adj_ = .531. The peak-to-peak amplitude did not differ significantly between congruent versus neutral conditions or incongruent versus neutral conditions, *t*(4) = −1.45, *p =* .222; *t*(4) = .48, *p =* .653.

## 4. Discussion

The aim of the present study was to investigate the neural mechanisms underlying the resolution of conflict in Spanish–English bilinguals with and without aphasia. We focused on a medial frontal component previously associated with crosslinguistic interference resolution during word retrieval and nonlinguistic conflict resolution. The expected behavioral facilitation and interference effects were present across both the linguistic PWI and nonlinguistic spatial Stroop tasks. EEG results revealed a medial frontal negative component peaking between 400–600 ms post-stimulus onset across the PWI and spatial Stroop tasks in healthy controls. The same medial frontal component peaked around −500 ms before the response in the PWI task and around −200 ms before the response in the nonlinguistic task in healthy controls. A similar component was present in bilinguals with aphasia post-stimulus onset and pre-response in the spatial Stroop task but was absent in the PWI task. These findings suggest that the medial frontal conflict resolution mechanism that is shared between linguistic and nonlinguistic tasks is selectively impaired in linguistic processing following stroke to the left hemisphere language regions.

### 4.1. Behavioral findings

Behavioral results revealed the expected facilitation and interference effects in both the linguistic and nonlinguistic tasks for accuracy rates and reaction times in the control participants and in bilinguals with aphasia. The bilinguals with aphasia were overall slower and less accurate than the control participants in the linguistic PWI task, as expected given their aphasia diagnosis and as previously reported (Calabria et al., 2019; Piai et al., 2016). Bilinguals with aphasia showed larger within-language facilitation effects than controls on accuracy in the PWI task. Thus, superimposed text in the identity condition contributed more greatly to Spanish picture naming success for bilinguals with aphasia compared to the control participants. Individuals with stroke-induced brain lesions, and especially with lesions in the left PFC, have been shown to be particularly affected by lexical interference in the semantic version of the PWI task (Piai et al., 2016). In other words, if the superimposed word is different than the picture name (or a string of Xs in the Piai et al.’s study), whether it is semantically related or unrelated, their performance is particularly more error prone. Differences between unrelated and related conditions can therefore be hard to observe given this previously observed general difficulty to discard the distractor word when it does not match the picture name. Given this, the increased facilitation effect we observe in the current study could be due to the unrelated condition being much more difficult for bilinguals with aphasia than for the control participants. This is indeed what we observed in the current study as the average accuracy rate was 47% (*SD* = 20.2) for bilinguals with aphasia in the unrelated condition and 41.6% (*SD* = 22.6) in the false cognate condition. These averages in bilinguals with aphasia were substantially lower compared to controls (UR: 92% accuracy, *SD* = 8.9; FC: 88% accuracy, *SD* = 9.5). The crosslinguistic PWI interference effect was not larger in bilinguals with aphasia than in control participants. This could also be linked to the fact that both the unrelated and false cognate conditions are much more difficult for bilinguals with aphasia than for control participants. Indeed, this very low performance in unrelated and false cognate trials could mask a potential increased crosslinguistic effect in bilinguals with aphasia compared to control participants, in a similar way as how the very low performance in unrelated and semantically related trials masks the semantic interference effect in individuals with left PFC stroke-induced lesions reported in Piai et al. (2016).

There was no group difference in nonlinguistic spatial Stroop performance accuracy between groups. This is consistent with some of the previous research comparing participants with and without aphasia on the traditional color Stroop paradigm (Green et al., 2010; Scott & Wilshire, 2010). However, other studies have reported lower accuracy in bilinguals with aphasia compared to healthy controls on the same color Stroop task (de Bruijn et al., 2014; Faroqi-Shah et al., 2018; Wiener et al., 2004). Zakariás et al. (2013) found differences between bilinguals with and without aphasia on a nonlinguistic spatial Stroop task similar to the spatial Stroop task employed in the current study, with the addition of two spatial positions (i.e., top and bottom) and directions (i.e., up and down). The discrepancy in findings in identified group differences on the Stroop task may in part be due to an increase in the number of response options available in the Zakariás et al., study (Zakariás et al., 2013) and the relatively lower degree of conflict resolution required in the current study. It is likely that our findings align more closely with studies where no group differences were found (Green et al., 2010; Scott & Wilshire, 2010). It is also possible that impairment patterns in nonlinguistic interference resolution are more variable than impairments in linguistic interference resolution in bilinguals with aphasia as conflict resolution mechanisms have been found to only partially overlap across domains (e.g., Mendoza et al., 2021) and the primary locus of impairment in aphasia is in the linguistic domain. Altogether, behavioral findings suggest group differences on the linguistic task, with a focus on group differences in the area of within-language facilitation, while the groups with and without aphasia are more closely aligned in performance on the nonlinguistic spatial Stroop task.

### 4.2. EEG findings

In the picture word interference task, EEG results revealed a negative-going component at the medial frontal electrode FCz peaking on average between 400 and 500 ms post-stimulus onset and around 500 ms prior to the response across conditions in healthy controls. Given that the average behavioral response time was at 1047 ms in control participants, these medial frontal components observed time-locked to the stimulus and to the response are likely to reflect two sides of the same coin. In contrast, in bilinguals with aphasia, this medial frontal component was absent. The medial frontal component reported here has been reported in previous overt picture-naming studies following stimulus-onset (Hirschfeld et al., 2008; Blackford et al., 2012; Riès et al., 2013) and preceding vocal onset (Pinet & Nozari, 2023; Riès et al., 2013) and has been associated with response selection within language (Pinet & Nozari, 2023; Riès et al., 2013) and outside of language (Vidal et al., 2011). A recent study associated a similar medial frontal component with crosslinguistic interference resolution prior to motor response in young Spanish–English bilinguals (Mendoza et al., 2021). Absence of this component in bilinguals with aphasia is consistent with their low performance on unrelated and false cognate trials in the current study, where alternatives must be removed from consideration during the lexical selection process.

In the nonlinguistic spatial Stroop task, EEG results for control participants revealed a stimulus-locked negative-going component at the same electrode peaking in the same time window as in the linguistic task (400–500 ms) post stimulus, with a response-locked component that peaked on average 200 ms prior to the response. Given that the average reaction time was around 800 ms in the spatial Stroop task in controls, this medial frontal component is also likely reflecting the same mechanism but seen from different, that is, stimulus- and response-locked, angles.

An overlap in components across tasks in older adult controls may suggest the recruitment of a similar cognitive control mechanism used to resolve conflict across domains, as found in Mendoza et al., 2021 in young bilingual adults. A study using Laplacian transformation in a simple picture naming task found a component at FCz peaking around 300 ms post-stimulus onset and 250 ms pre-vocal onset in native French speakers (Riès et al., 2013). A comparable activity reported in an early MEG study (Salmelin et al., 1994) found a fronto-central component peaking approximately 450 ms following picture onset, before vocal onset. Studies investigating nonverbal conflict have found similar EEG negativities peaking between 300 and 500 ms post-stimulus onset in native English speakers in fronto-central recording sites (Hanslmayr et al., 2008; Naylor et al., 2012) and preceding response execution (Roger, 2009; Vidal et al., 2003; Vidal et al., 2011). Source localization analyses have associated this component with increased activity from the ACC (Hanslmayr et al., 2008; Szűcs & Soltész, 2010), which has been shown to be active in situations of high conflict (Barch et al., 2000; Kerns et al., 2004). Specifically, the dorsal ACC in the PFC has been associated with domain-general cognitive control and lexical selection (Braver, 2012; Piai et al., 2013; de Zubicaray et al., 2006).

In bilinguals with aphasia, performance on the nonlinguistic spatial Stroop task revealed a similar negative going component peaking at the same electrode and window as in the control participants post-stimulus onset between 400 and 500 ms, as well as approximately 200 ms pre-response. The absence of the medial frontal component in the linguistic PWI task, but not the nonlinguistic spatial Stroop task, suggests that the mechanism used to resolve conflict continues to be recruited despite disruption to the language system. Thus, the linguistic system’s access to this domain-general conflict resolution mechanism appears to be impaired following stroke-induced aphasia, while the mechanism itself continues to function in nonlinguistic tasks. These findings further support previous functional brain imaging research suggesting overlapping neural activation in the medial frontal cortex, and specifically the rostral ACC, between the semantic interference version of the PWI task and the Stroop and Simon tasks in native Dutch speakers (Piai et al., 2013).

### 4.3. Theoretical Implications

Our focus on a medial frontal ERP component highlights the central role of the underlying mechanism in the top-down regulation of control during crosslinguistic interference. However, it is important to consider this region as part of a broader network involved in language and cognitive control. Bilingual speakers activate additional regions outside the classical perisylvian language network as part of the bilingual language control network. According to the Adaptive Control Hypothesis, the ACC monitors crosslinguistic conflict and signals when control is needed (Green & Abutalebi, 2013). The PFC manages top-down control by suppressing the non-target language, while the parietal lobules support attentional shifting and language goal maintenance. Within the basal ganglia, the caudate nucleus supports language selection, particularly when multiple alternatives are present and based on context, whereas the putamen supports articulatory control during speech production (Abutalebi & Green, 2007, 2013, Abutalebi & Green, 2016). These regions overlap with those proposed in the Dual Mechanisms of Cognitive Control framework (Braver, 2012) in which the dorsal ACC is proposed to work in concert with the dorsolateral PFC to detect conflict and initiate appropriate control adjustments (Braver, 2012). Our findings contribute to these neurobiological frameworks by suggesting that bilingual response selection engages a medial frontal mechanism, as seen in young bilinguals in Mendoza et al. (2021), peaking prior to response onset in the PWI and nonlinguistic tasks. Previous research on language switching and language selection in bilinguals has revealed robust functional activity in the dACC/Pre-SMA (Abutalebi et al., 2007; Abutalebi et al., 2012; Abutalebi & Green, 2008; Branzi et al., 2015; Guo et al., 2011; Hosoda et al., 2012; Wang et al., 2007), further supporting the role of the medial frontal cortex in linguistic response selection.

Additionally, the medial PFC is known to be anatomically and functionally connected to perisylvian language regions through white matter pathways including the frontal aslant tract (FAT; Catena Baudo et al., 2023; Chernoff et al., 2018; La Corte et al., 2021). The medial frontal control mechanism appears to be selectively impaired during linguistic processing in our study potentially because of the disruption of connections between the medial PFC and the perisylvian cortex in our bilingual participants (see Table 4; La Corte et al., 2021). These are indeed commonly affected in individuals with stroke-induced aphasia (Nogles & Galuska, 2024). In our bilingual participants, the left precentral gyrus, SMA and the superior and middle frontal gyri were variably compromised in all four individuals with available lesion data. Moreover, critical FAT termination sites – including the left IFG (pars opercularis and triangularis) and the insula – were also affected in the majority of individuals. Damage to the left frontal cortex would explain why the medial PFC cannot perform its role in crosslinguistic conflict resolution. In contrast, the non-linguistic task may not recruit language-specific left perisylvian regions to the same extent as the linguistic task, which would explain the dissociation we observe. Therefore, these findings support the view that bilingual language control relies in part on brain regions responsible for conflict resolution within a domain-general executive control system and on language-specific regions for language processing.

### 4.4. Limitations and future directions

We identified the expected medial-frontal ERP in our linguistic and nonlinguistic tasks but its amplitude was not modulated by condition. Other studies investigating ERP correlates of conflict resolution have reported amplitude differences between related and unrelated conditions with and without Laplacian transformation (Anderson et al., 2022; Andras et al., 2023; Blackford et al., 2012; Mendoza et al., 2021). However, the number of control participants in the current study (n = 20) was larger than in our previous study (n = 12) that showed a significant crosslinguistic interference effect on ERPs (Mendoza et al., 2021). In this previous study, the linguistic paradigm used differed from the one used here as it was a picture-word matching task between the picture and the superimposed word instead of a naming task in which the distractor word had to be ignored. A larger number of participants could be needed in the naming version of this paradigm, which will have to be investigated in future studies. While future work in this area is needed, the current findings provide important early evidence for the nature of aphasia-related impairment in cognitive processes associated with lexical retrieval for naming.

Lastly, in this study, we could not account for interindividual variability in cognitive skills or relative language proficiency, given our relatively small sample size. While aphasia is traditionally viewed as a language-specific disorder, impairments supporting cognitive domains are frequently observed (Martin & Reilly, 2012; Murray, 2012). Including additional neuropsychological assessments that capture a more comprehensive cognitive-linguistic profile may contribute to further understanding how much cognitive processes (i.e., attention and working memory) are engaged or involved in an impaired language system (Silkes & Anjum, 2021). Further, performance on crosslinguistic and nonlinguistic cognitive control tasks have been shown to be modulated by language proficiency and use in bilingual speakers (Kheder & Kaan, 2021; van den Noort et al., 2019). Given the documented importance of these variables on word retrieval abilities in bilinguals (Marian & Hayakawa, 2020), future studies will need to consider these variables to provide a more complete understanding of the underlying neural mechanisms at play. In addition, given the relatively high linguistic demands of the MoCA that was used in the current study, future studies may want to consider using the cognitive assessment scale for stroke patients (CASP) instead to assess cognitive impairment in this population (Barnay et al., 2014).

## 5. Conclusions

The current study confirms previous findings from Mendoza et al. (2021) pointing to a medial frontal mechanism involved in crosslinguistic and nonlinguistic conflict resolution in bilinguals. In the current study, we extend these findings to an overt naming task and to the study of aphasia. Our findings are consistent with studies investigating conflict resolution within language that have associated the medial PFC during naming with response selection and conflict resolution processes (Alario et al., 2006; Piai et al., 2013; Ridderinkhof et al., 2011). Evidence from bilingual individuals with aphasia in the current study indicates that the medial PFC’s involvement is selectively impaired in language processing. This is likely caused by a disconnection between the medial PFC from other regions in the bilingual language control network and perisylvian language regions following MCA infarct. Our results are consistent with the proposal that a medial frontal domain-general mechanism may be engaged to resolve conflict in and outside of language but its interactions with more language-specific regions appear critical to support linguistic and not non-linguistic conflict.

## Supporting information

Andrade et al. supplementary material Andrade et al. supplementary material

## Data Availability

Please contact the corresponding author for access to the deidentified data.

## Supplementary material

The supplementary material for this article can be found at http://doi.org/10.1017/S1366728925100461.

## Long descriptions

### Table 1. Long description

The table is organized into columns for Controls (n = 21) and five individual patients: P T 1, P T 4, P T 5, P T 7, and P T 9.

Key demographic data includes:

* Age: Controls mean 52.2; Patients range from 38 to 77.

* Gender: Controls 16 F, 5 M; Patients 4 F, 1 M.

* Years of education: Controls mean 16; Patients range from 13 to 18.

* Age of acquisition - Spanish: Controls mean 1.0; Patients range from 0 to 5.

* Age of acquisition - English: Controls mean 11.5; Patients range from 0 to 16.

* Current language dominance: Controls mean -0.065 (English dominant); Patients range from 0.35 (Spanish dominant) to -0.60 (English dominant).

Clinical data for patients includes:

* Time since stroke: 16 to 64 months.

* Aphasia severity: Mostly mild mixed expressive and receptive, with P T 7 being moderate.

* Cognition severity: Ranges from mild to moderate cognitive impairment.

* Apraxia severity: Ranges from none to moderate apraxia of speech.

* Type of stroke: All five patients experienced a hemorrhage.

* Localization: All involve the left hemisphere, including frontal, parietal, and temporal lobes, as well as the insula.

Navigate back to Table 1..

### Table 2. Long description

The table is organized into eight columns. The first column lists the assessments: M O C A, C L Q T dash S C, and C L Q T dash D M. The second column lists the maximum raw scores for these tests as 30, 12, and 6 respectively. The third column provides the control group mean, standard deviation, and range as proportions: M O C A is .87 (S D .09; range .66 to 1), C L Q T dash S C is .99 (S D .03; range .91 to 1), and C L Q T dash D M is .87 (S D .11; range .66 to 1).

The remaining five columns show individual proportions for patients P T 1, P T 4, P T 5, P T 7, and P T 9:

* M O C A scores: .53, .47, .67, .82, and .67.

* C L Q T dash S C scores: D N T (Did Not Test), 0, 1.0, 1.0, and 1.0.

* C L Q T dash D M scores: D N T, .83, .67, 1.0, and .67.

Navigate back to Table 2..

### Table 3. Long description

The table contains 19 rows of assessment data. The columns are organized by participant (P T 1, P T 4, P T 5, P T 7, P T 9), each subdivided into E N G and S P N.

* M I N T (Max 68): Scores range from .19 to .86 across participants.

* B A T - B Simple and semi-complex demands (Max 5): Most participants scored 1.0, with P T 1 S P N at .8.

* B A T - B Verbal auditory discrimination (Max 18): Scores range from .72 to 1.0.

* B A T - B Semantic categories (Max 5): Scores range from .4 to 1.0.

* B A T - B Repetition (Max 60): Scores range from .78 to 1.0.

* B A T - B Verbal fluency (Max N A): Raw scores range from 4 to 30.

* B A T - B Listening comprehension (Max 5): Scores range from 0 to 1.0.

* B A T - B Reading comprehension words (Max 10): Scores range from .8 to 1.0.

* B A T - B Reading comprehension sentences (Max 10): Scores range from .4 to 1.0.

* B A T - C Word recognition (Max 1.0): All participants scored 1.0.

* B A T - C Translation of words (Max 10): Scores range from .2 to 1.0.

* A B A - 2 Diadochokinetic rate (Thresholds: 26 plus N I, 7 to 25 M I): Scores range from 7 to 32.

* A B A - 2 Increasing word length a (Thresholds: less than 1 N I, 2 to 4 M I, 5 to 7 M O, 8 plus S I): Scores range from -1 to 16.

* A B A - 2 Increasing word length b (Thresholds: less than 1 N I, 2 M I, 3 to 5 M O, 6 plus S I): Scores range from -4 to 11; P T 7 S P N is D N T.

* A B A - 2 Limb apraxia (Threshold: 44 to 50 N I): Scores range from 41 to 50.

* A B A - 2 Oral apraxia (Threshold: 44 to 50 N I): Scores range from 45 to 50.

* A B A - 2 Latency and utterance time (Thresholds: 0 to 15 N I, 16 to 55 M I): Scores range from 5 to 56.

* A B A - 2 Repeated trials (Thresholds: 28 to 30 N I, 16 to 27 M I, 5 to 15 M O): Scores range from 7 to 30.

* A B A - 2 Inventory of articulation characteristics (Max N A): Scores range from 0 to 12.

Navigate back to Table 3..

### Figure 1. Long description

The multi-panel display consists of two rows of brain scans.

Top Row: Ten axial slices numbered 5 through 50 in increments of 5. These slices show the horizontal cross-sections of the brain from the base toward the top. On the far right is a sagittal view of the brain with horizontal blue lines indicating the position of these axial slices.

Bottom Row: Ten coronal slices numbered -13 through 32 in varying increments. These slices show vertical cross-sections from the front to the back of the brain. On the far right is a sagittal view with vertical blue lines indicating the position of these coronal slices.

Data Visualization:

* Colored overlays on the left hemisphere of each slice represent individual lesions for four participants: P T 1 (dark red), P T 4 (magenta), P T 7 (purple), and P T 9 (teal).

* A light pink color indicates the area of lesion overlap where all four participants share common brain damage.

* The lesions are concentrated in the left frontal and temporal regions, including the Broca area and surrounding white matter tracts.

* A legend at the bottom identifies the color coding for P T 1, P T 4, P T 7, P T 9, and the Lesion overlap (n=4).

Navigate back to Figure 1..

### Table 4. Long description

The table lists voxel damage percentages across anatomical classifications: Frontal, Prefrontal, Parietal, Subcortical, and Temporal.

Frontal regions include:

* Left precentral gyrus: All 93%, P T 1 92%, P T 4 85%, P T 7 13%, P T 9 17%.

* Left supplemental motor area (S M A): All 50%, P T 1 47%, P T 4 15%, P T 7 less than 1%, P T 9 no damage.

* Left dorsal cingulate cortex (d A C C): All 30%, P T 1 5%, P T 4 23%, P T 7 9%, P T 9 no damage.

* Left rolandic operculum: All 91%, P T 1 82%, P T 4 66%, P T 7 71%, P T 9 31%.

Prefrontal regions include:

* Left anterior cingulate cortex (A C C): All less than 1%, P T 4 less than 1%, others no damage.

* Left superior frontal gyrus (S F G): All 45%, P T 1 45%, P T 4 21%, others no damage.

* Left middle frontal gyrus (M F G): All 52%, P T 1 47%, P T 4 40%, P T 7 less than 1%, P T 9 1%.

* Left inferior frontal gyrus, pars opercularis (I F G): All 90%, P T 1 89%, P T 4 68%, P T 7 16%, P T 9 39%.

* Left inferior frontal gyrus, pars triangularis (I F G): All 47%, P T 1 38%, P T 4 28%, P T 7 less than 1%, P T 9 5%.

Parietal regions include:

* Left inferior parietal cortex: All 75%, P T 1 67%, P T 4 5%, P T 7 12%, P T 9 8%.

* Left angular gyrus: All 54%, P T 1 4%, P T 7 51%, P T 9 5%.

* Left supramarginal gyrus: All 71%, P T 1 41%, P T 4 1%, P T 7 57%, P T 9 30%.

* Left superior parietal: All 20%, P T 1 20%, P T 4 less than 1%.

Subcortical regions include:

* Left insula: All 82%, P T 1 81%, P T 4 26%, P T 7 42%, P T 9 21%.

* Left caudate: All 20%, P T 1 less than 1%, P T 4 18%, P T 7 7%.

* Left putamen: All 55%, P T 1 49%, P T 4 6%, P T 7 40%, P T 9 less than 1%.

* Left thalamus: All 21%, P T 1 1%, P T 7 20%.

* Left pallidum: All 20%, P T 1 20%, P T 7 4%.

Temporal regions include:

* Left Heschl’s gyrus: All 99%, P T 1 56%, P T 4 45%, P T 7 93%, P T 9 32%.

* Left middle temporal gyrus (M T G): All 25%, P T 1 5%, P T 7 20%, P T 9 less than 1%.

* Left superior temporal gyrus (S T G): All 68%, P T 1 24%, P T 4 5%, P T 7 48%, P T 9 9%.

Navigate back to Table 4..

### Table 5. Long description

The table is organized into five columns. The first column lists the participant groups, while the remaining four columns are grouped under two main tasks: P W I and Spatial Stroop. Each task includes two metrics: Mean accuracy rate (S D; range) and Mean reaction time (S D; range).

* Controls (n = 21):

- P W I: 91.79% accuracy (6.49%; 80–99%); 1047 ms reaction time (323 ms; 774–1321 ms).

- Spatial Stroop: 94.62% accuracy (6.6%; 77–100%); 585 ms reaction time (180 ms; 277–2624 ms).

* Bilinguals with aphasia (n = 5):

- P W I: 59.33% accuracy (18%; 33–80%); 1419 ms reaction time (435 ms; 1218–1681 ms).

- Spatial Stroop: 97.91% accuracy (2.36%; 94–100%); 755 ms reaction time (328 ms; 298–2804 ms).

* Individual Patient Data (P W I accuracy, P W I reaction time, Spatial Stroop accuracy, Spatial Stroop reaction time):

- P T 1: 70.24%, 1470 ms, 99.76%, 621 ms.

- P T 4: 54.05%, 1681 ms, 98.10%, 783 ms.

- P T 5: 79.52%, 1218 ms, 99.29%, 543 ms.

- P T 7: 32.86%, 1366 ms, 93.84%, 621 ms.

- P T 9: 60.00%, 1419 ms, 98.56%, 1188 ms.

Navigate back to Table 5..

### Figure 2. Long description

Panel A is a box plot of P W I Accuracy Rate percentage. The y-axis ranges from 0 to 100. Controls show high accuracy above 75 percent across False Cognate (orange), Unrelated (grey), and Identity (blue) conditions. Bilinguals with Aphasia show significantly lower accuracy, particularly in the False Cognate condition (median near 40 percent) and Unrelated condition (median near 50 percent), with Identity being the highest (median near 85 percent).

Panel B is a box plot of P W I Reaction Time in m s. The y-axis ranges from 0 to 3000. Controls have faster, tighter reaction times clustered around 1000 m s. Bilinguals with Aphasia show slower reaction times with higher variability, with medians between 1200 and 1600 m s across all conditions.

Panel C is a box plot of Spatial Stroop Accuracy Rate percentage. The y-axis is zoomed from 80 to 100. Both groups perform well, though Controls show more variance in the Incongruent condition. Bilinguals with Aphasia maintain high accuracy above 90 percent across Incongruent, Neutral, and Congruent conditions.

Panel D is a box plot of Spatial Stroop Reaction Time in m s. The y-axis ranges from 0 to 3000. Controls are faster with medians below 1000 m s. Bilinguals with Aphasia are slightly slower but more consistent than in the P W I task, with medians also remaining below 1000 m s. In all panels, horizontal lines within boxes represent medians and dots represent outliers.

Navigate back to Figure 2..

### Figure 3. Long description

A four-panel multi-plot showing Laplacian-transformed E R P waveforms at electrode F C z. Note that negative amplitude is plotted upward on the y-axis.

Panel A: P W I Stimulus-Locked. The x-axis shows Time in m s from minus 400 to 1000. The y-axis shows Amplitude in microvolts per centimeter-squared from 20 to minus 20. Three conditions are plotted: False Cognate (orange), Unrelated (gray), and Identity (blue). A vertical dashed line marks stimulus onset at 0 m s. A yellow shaded region between 200 and 400 m s highlights a negative peak. Insets show three scalp topographies for each condition between 464 and 516 m s, with blue indicating negative activity at the fronto-central region.

Panel B: P W I Response-Locked. The x-axis ranges from minus 600 to 800 m s. Waveforms for the same three conditions converge near 0 m s. A yellow shaded region is highlighted between minus 600 and minus 400 m s. Topographies for the minus 500 to minus 448 m s window show central blue activity.

Panel C: Spatial Stroop Stimulus-Locked. Conditions include Incongruent (orange), Neutral (gray), and Congruent (blue). A prominent negative deflection occurs after 200 m s, highlighted in yellow. Topographies for 596 to 648 m s show concentrated blue activity at the top of the scalp map for the Incongruent condition.

Panel D: Spatial Stroop Response-Locked. The x-axis ranges from minus 600 to 800 m s. All conditions show a sharp negative peak at 0 m s. A yellow shaded region spans minus 400 to minus 200 m s. Topographies for minus 208 to minus 156 m s show blue activity concentrated in the fronto-central area for the Incongruent condition.

Navigate back to Figure 3..

### Figure 4. Long description

A four-panel set of line graphs labeled A through D. All graphs plot Amplitude in microvolts per centimeter-squared on the y-axis against Time in milliseconds on the x-axis. Note that negative amplitude is plotted upward.

* Panel A: P W I Stimulus-Locked waveforms at electrode F C z. Three lines represent False Cognate (orange), Unrelated (gray), and Identity (blue) conditions. A vertical dashed line marks time zero. A yellow shaded region spans approximately 180 to 380 milliseconds. Inset topographies for the three conditions show blue-toned negativity concentrated in the fronto-central region.

* Panel B: P W I Response-Locked waveforms. The orange False Cognate line shows a prominent negative peak around 350 milliseconds post-response. Topographies show a strong central blue focal point for False Cognates compared to Unrelated and Identity conditions.

* Panel C: Spatial Stroop Stimulus-Locked waveforms. Lines represent Incongruent (orange), Neutral (gray), and Congruent (blue). A yellow shaded region spans 180 to 380 milliseconds. The waveforms show a negative deflection peaking around 200 milliseconds. Topographies show red-toned positivity in the posterior regions and blue-toned negativity in the frontal regions.

* Panel D: Spatial Stroop Response-Locked waveforms. A yellow shaded region is highlighted between negative 400 and negative 200 milliseconds. The waveforms converge toward zero at the time of response. Topographies show a diffuse distribution of activity across the scalp.

Navigate back to Figure 4..
