# Supplementary material for: Brain dynamics of crosslinguistic interference resolution in Spanish–English bilinguals with and without aphasia
Source: Biling (Camb Engl). 2025 Sep 5;29(4):872–90. doi: 10.1017/S1366728925100461 (PMC12752478; doi:10.1017/S1366728925100461)
Supplement: Andrade et al. supplementary material [file S1366728925100461sup001.docx]

**Appendix**


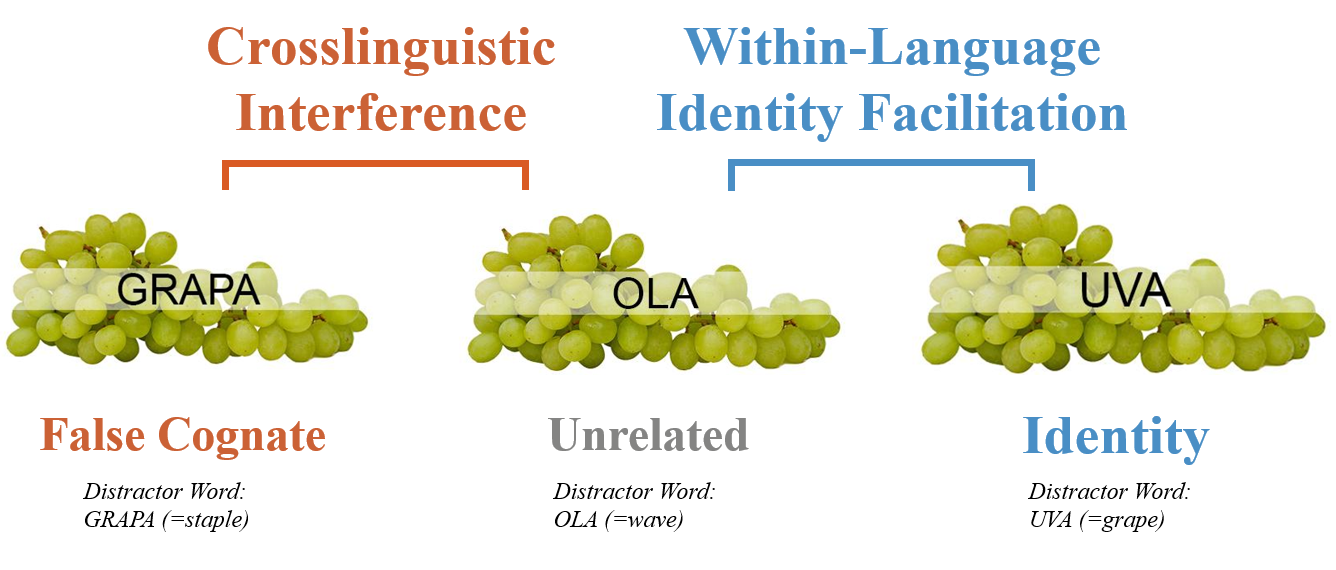


**Figure A1.** Example stimuli of the false cognate condition, unrelated condition, and identity condition, where the Spanish naming target was “uva.” Crosslinguistic overlap exists in the false cognate condition but not in the unrelated condition, with superimposed words (e.g., GRAPA) resembling the translation equivalent of the Spanish naming target (“grapes’) but mapping onto a different meaning (“staple”). Within-language cueing existed in the identity but not the unrelated conditions, with the superimposed word (e.g., UVA) priming the Spanish naming target.

**
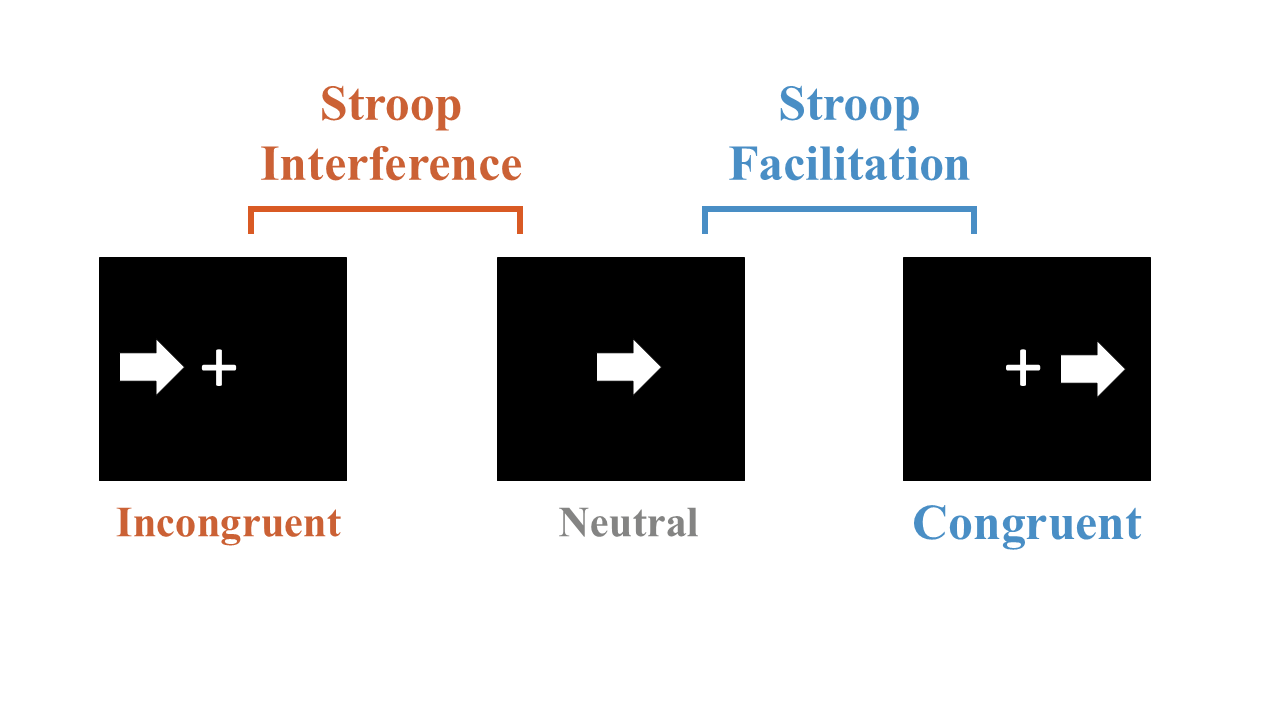
**

**Figure A2.** Example stimuli of the incongruent condition, neutral condition, and congruent condition in the spatial Stroop task. Stroop interference exists in the incongruent condition but not in the neutral condition. Stroop facilitation exists in the congruent condition but not the neutral condition.

**Table A3.** Comprehensive list of stimuli used in the PWI task across conditions (e.g., false cognate, unrelated, and identity). Distractor words presented in Spanish over the picture. English translations of stimuli provided, but not presented during experiment.

|  | **Picture:**  **Target Name** | | **Distractor:**  **False Cognate** | | | **Distractor:**  **Unrelated** | | **Distractor:**  **Identity** | |
| --- | --- | --- | --- | --- | --- | --- | --- | --- | --- |
|  | **Presented Picture** | *English Translation* | | **Presented Word** | *English Translation* | **Presented**  **Word** | *English Translation* | **Presented Word** | *English Translation* |
| 1. | Aceite | *oil* | | OLA | *wave* | CANDADO | *lock* | ACEITE | *oil* |
| 2. | Alombra | *carpet* | | CARPETA | *folder* | PAN | *bread* | ALOMBRA | *carpet* |
| 3. | Cabra | *boat* | | GOTA | *raindrop* | PARED | *wall* | CABRA | *goat* |
| 4. | Chancla | *sandal* | | SANDÍA | *watermelon* | PLUMA | *feather* | CHANCLA | *sandal* |
| 5. | Ciruela | *plum* | | PLUMA | *feather* | GRAPA | *staple* | CIRUELA | *plum* |
| 6. | Desfile | *parade* | | PARED | *wall* | GOTA | *raindrop* | DESFILE | *parade* |
| 7. | Flecha | *arrow* | | ARROZ | *rice* | MAÍZ | *corn* | FLECHA | *arrow* |
| 8. | Laberinto | *maze* | | MAÍZ | *corn* | ARROZ | *rice* | LABERINTO | *maze* |
| 9. | Lluvia | *rain* | | REINA | *queen* | BURRO | *donkey* | LLUVIA | *rain* |
| 10. | Pizarra | *board* | | BURRO | *donkey* | REINA | *queen* | PIZARRA | *board* |
| 11. | Sanguijuela | *leech* | | LECHE | *milk* | BOCA | *mouth* | SANGUIJUELA | *leech* |
| 12. | Sartén | *pan* | | PAN | *bread* | CARPETA | *folder* | SARTÉN | *pan* |
| 13. | Tarta | *pie* | | PIE | *foot* | LADRILLO | *brick* | TARTA | *pie* |
| 14. | Uva | *grape* | | GRAPA | *staple* | OLA | *wave* | UVA | *grape* |
| 15. | Vela | *candle* | | CANDADO | *lock* | PIE | *foot* | VELA | *candle* |
| 16. | Cucharon | *ladle* | | LADRILLO | *brick* | SANDÍA | *watermelon* | CUCHARON | *ladle* |
| 17. | Caja | *box* | | BOCA | *mouth* | LECHE | *milk* | CAJA | *box* |
| 18. | Tacón | *heel* | | HILO | *string* | PECHO | *chest* | TACÓN | *heel* |
| 19. | Durazno | *peach* | | PECHO | *chest* | HORNO | *oven* | DURAZNO | *peach* |
| 20. | Chícharo | *pea* | | PEZ | *fish* | HILO | *string* | CHÍCHARO | *pea* |
| 21. | Cuerno | *horn* | | HORNO | *oven* | PEZ | *fish* | CUERNO | *horn* |
| 22. | Hilo | *string* | | - | - | DURAZNO | *peach* | HILO | *string* |
| 23. | Pecho | *chest* | | - | - | CUERNO | *horn* | PECHO | *chest* |
| 24. | Pez | *fish* | | - | - | TACÓN | *heel* | PEZ | *fish* |
| 25. | Carpeta | *folder* | | - | - | ACEITE | *oil* | CARPETA | *folder* |
| 26. | Gota | *raindrop* | | - | - | CHANCLA | *sandal* | GOTA | *raindrop* |
| 27. | Sandía | *watermelon* | | - | - | DESFILE | *parade* | SANDÍA | *watermelon* |
| 28. | Pared | *wall* | | - | - | LLUVIA | *rain* | PARED | *wall* |
| 29. | Arroz | *rice* | | - | - | CABRA | *goat* | ARROZ | *rice* |
| 30. | Maíz | *corn* | | - | - | SANGUIJUELA | *leech* | MAÍZ | *corn* |
| 31. | Reina | *queen* | | - | - | UVA | *grape* | REINA | *queen* |
| 32. | Burro | *donkey* | | - | - | TARTA | *pie* | BURRO | *donkey* |
| 33. | Leche | *milk* | | - | - | PIZARRA | *board* | LECHE | *milk* |
| 34. | Pan | *bread* | | - | - | LABERINTO | *maze* | PAN | *bread* |
| 35. | Grapa | *staple* | | - | - | CAJA | *box* | GRAPA | *staple* |
| 36. | Boca | *mouth* | | - | - | CIRUELA | *plum* | BOCA | *mouth* |
| 37. | Pie | *foot* | | - | - | CUCHARON | *ladle* | PIE | *foot* |
| 38. | Candado | *lock* | | - | - | FLECHA | *arrow* | CANDADO | *lock* |
| 39. | Ladrillo | *brick* | | - | - | ALFOMBRA | *carpet* | LADRILLO | *brick* |
| 40. | Ola | *wave* | | - | - | SARTÉN | *pan* | OLA | *wave* |
| 41. | Pluma | *feather* | | - | - | VELA | *candle* | PLUMA | *feather* |
| 42. | Horno | *oven* | | - | - | CHÍCARO | *pea* | HORNO | *oven* |
